# Integrative Analysis Uncover the Effects and Multi‐Omics Features of Thigh Muscle Fat Infiltration

**DOI:** 10.1111/acel.70690

**Published:** 2026-08-31

**Authors:** Yiwei Zhang, Qin Dang, Xizeng Zong, Haowei Chen, Simin Wen, Siqi Xu, Kunyan Wang, Xiaoshuai Wang, Jianwei Zhu, Zhaohua Zhu, Changhai Ding, Guangfeng Ruan, Yan Zhang

**Affiliations:** ^1^ Clinical Research Center Zhujiang Hospital, Southern Medical University Guangzhou Guangdong China; ^2^ Clinical Research Centre, School of Medicine The Second Affiliated Hospital of South China University of Technology (Guangzhou First People's Hospital) Guangzhou Guangdong China; ^3^ Department of Rehabilitation Medicine The Third Affiliated Hospital, Southern Medical University Guangzhou Guangdong China; ^4^ Department of Orthopedics, School of Medicine The Second Affiliated Hospital of South China University of Technology (Guangzhou First People's Hospital) Guangzhou Guangdong China; ^5^ Clinical Research Centre Beijing Tsinghua Changgung Hospital, Tsinghua Medicine, Tsinghua University Beijing China; ^6^ Menzies Institute for Medical Research University of Tasmania Hobart Australia

**Keywords:** genome‐wide association study, health‐related outcomes, lifestyle factors, multi‐omics, thigh muscle fat infiltration

## Abstract

The health impacts and underlying biological pathways of thigh muscle fat infiltration (TMFI) remain incompletely understood. In this study, we analyzed TMFI measured by magnetic resonance imaging in 55,120 UK Biobank participants and found that higher TMFI was significantly associated with all‐cause mortality as well as with all major system‐specific diseases examined (*p* values ranged from 2.50 × 10^−88^ to 9.97 × 10^−04^). TMFI also mediated the effects of lifestyle factors on health‐related outcomes, with mediation proportions ranging from 6.7% to 71.7%. A genome‐wide association study (GWAS) identified 79 lead single nucleotide polymorphisms (SNPs) linked to TMFI, and the polygenic risk score for TMFI was significantly associated with mortality and all incident diseases across examined organ systems in an independent subset of UK Biobank participants of European ancestry who were not included in the TMFI GWAS (*n* = 362,286, all *p* < 0.05). Gene–drug interactions identified multiple drugs that could potentially modulate TMFI. Analysis of single‐cell transcriptomic data indicated that myogenic cells were strongly linked to TMFI (*p* = 7.08 × 10^−08^). Summary‐data‐based Mendelian randomization and Transcriptome‐Wide Association Study analyses revealed numerous genes whose expression in specific tissues was associated with TMFI. Proteomic and metabolomic profiling uncovered a broad array of circulating biomarkers associated with TMFI, many of which mediated the effects of modifiable factors and genetic risk on TMFI. Overall, our results highlight the biological relevance of TMFI to human health and provide insights into the multi‐omics mechanisms underlying TMFI, identifying potential targets for interventions.

## Graphical abstract

Thigh muscle fat infiltration (TMFI), estimated from MRI, is associated with increased mortality and diseases affecting multiple organ systems and mediates the effects of lifestyle factors on health. Multi‐omics analyses delineate the genetic, cellular, transcriptional, proteomic, and metabolic architecture of TMFI.

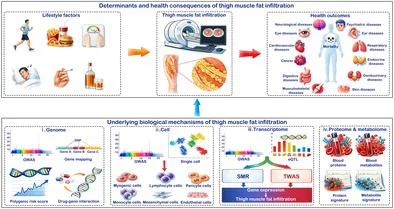

## 1 Introduction

Skeletal muscle is one of the largest organs in the human body, accounting for approximately 40% of total body mass (Duranti 2025). In addition to locomotion, posture maintenance, and force generation (Groeneveld 2024; Brooks et al. 2023), the overall health of skeletal muscle is critical for systemic metabolic homeostasis (Hulett et al. 2022; Lin et al. 2023). Specifically, higher muscle quality is associated with a lower risk of chronic conditions, including cardiovascular disease, diabetes, chronic kidney disease, and obesity (Zhou et al. 2023; Jun et al. 2023). Strategies to improve muscle quality are increasingly recognized as core components of chronic disease management, highlighting the need to clarify underlying mechanisms that support effective interventions. The thigh muscles are a central focus in studies of muscle quality due to their large contribution to total skeletal muscle mass and their essential roles in standing, walking, and load‐bearing activities (Fuchs et al. 2023).

During physical deconditioning and aging, skeletal muscle is progressively replaced by adipose tissue (Marcus et al. 2010). Thigh muscle fat infiltration (TMFI) refers to the ectopic accumulation of fat within thigh skeletal muscle. At the microscopic level, fat accumulates both extracellularly as adipocytes within the interstitial spaces between muscle fascicles and fibers and intracellularly as lipid droplets within myofibers, predominantly in subsarcolemmal and intermyofibrillar regions (Miljkovic and Zmuda 2010; Hausman et al. 2014; Bosma 2016). Such fat accumulation compromises muscle quality, leading to reduced strength, impaired mobility, and adverse metabolic alterations (Wang et al. 2024). Accordingly, quantification of TMFI has attracted considerable attention, with magnetic resonance imaging (MRI) regarded as a highly sensitive and noninvasive technique for this purpose (Sabatino et al. 2024; Janssen et al. 2016). Accumulating evidence indicates that increased TMFI is closely linked to the onset or progression of multiple diseases, including knee and hip osteoarthritis (Weng et al. 2025), diabetes (Huang et al. 2023), chronic kidney disease (Kim et al. 2024), and chronic obstructive pulmonary disease (Persson et al. 2022). These findings underscore the clinical and epidemiological importance of TMFI as an independent marker of thigh muscle quality.

Despite some progress in TMFI research over recent years, key knowledge gaps persist. Most studies have focused on TMFI with respect to single diseases, while comprehensive evaluations of its health implications are still lacking. In parallel, how lifestyle factors influence TMFI and subsequent health outcomes requires further exploration. Moreover, genome‐wide association studies (GWAS) investigating TMFI remain scarce, and its genetic characteristics are poorly understood. The specific cell types and molecular mechanisms contributing to TMFI have yet to be fully elucidated, and systematic analyses of circulating proteins and metabolites associated with TMFI are also absent. If these gaps can be addressed, it would advance our understanding of the relationship between TMFI and overall health, as well as the biological basis of TMFI. This knowledge could also inform the development of targeted interventions aimed at restoring thigh muscle quality and ultimately improving clinical outcomes.

Using data from the UK Biobank (UKB), this study delineates the associations of TMFI with all‐cause mortality and diseases across multiple organ systems. This study further demonstrates the mediating role of TMFI in linking lifestyle factors to diverse health outcomes. Crucially, this study elucidated the potential cellular and molecular mechanisms underlying TMFI by characterizing associated genetic variants, cell types, gene expression, and circulating proteins and metabolites. Together, these findings establish the biological relevance of TMFI and provide mechanistic insights that may guide future strategies for its prevention and intervention.

## 2 Results

### 2.1 TMFI Associated With Mortality and Diseases Across Multiple Systems

**FIGURE 1:**
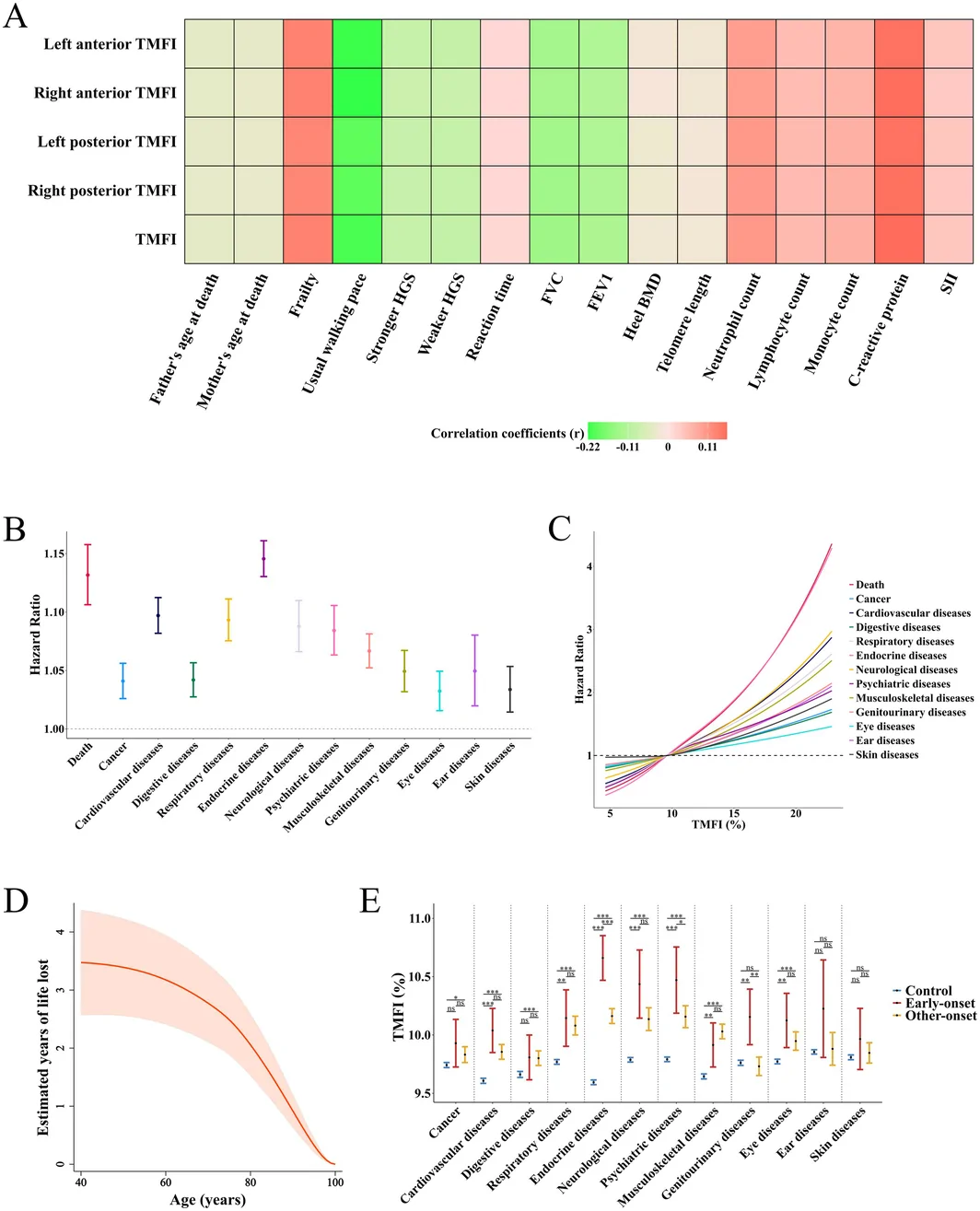
Thigh muscle fat infiltration associated with multisystem health outcomes. (A) Correlation coefficients of partial correlations of thigh muscle fat infiltration (TMFI) with aging‐related biomarkers and inflammation biomarkers. (B) Forest plot depicting hazard ratios for associations of TMFI with mortality and the incidences of multiple diseases. (C) The restricted cubic spline curves for the associations of TMFI with mortality and the incidences of multiple diseases. (D) Estimated years of life lost in the highest versus lowest quartile of TMFI. (E) TMFI levels were compared across early‐onset (top 10% youngest at disease diagnosis), other‐onset (all other patients not in the top 10% youngest), and disease‐free groups among diseases. The data are shown as estimated marginal means with 95% confidence intervals. Pairwise comparisons of estimated marginal means of TMFI were performed using the Emmeans test. Two‐sided *p* values from Emmeans tests were adjusted for multiple comparisons using the Benjamini‐Hochberg (BH) procedure. *, BH‐corrected *p* value < 0.05; **, BH‐corrected *p* value < 0.01; ***, BH‐corrected *p* value < 0.001. BMD, bone mineral density; FEV1, forced expiratory volume in 1‐s; FVC, forced vital capacity; HGS, hand grip strength; ns, nonsignificant; SII, systemic immune inflammation index; SRT, speech reception threshold; TMFI, thigh muscle fat infiltration.

Partial correlation analyses revealed significant associations of left anterior TMFI, right anterior TMFI, left posterior TMFI, right posterior TMFI, and total TMFI with parental lifespan, aging indicators and inflammatory indicators (Figure 1A, Table S1). Survival analysis indicated that TMFI in these four regions, as well as total TMFI, was significantly positively associated with all‐cause mortality and the incidence of various system‐specific diseases in a dose‐dependent manner (Figure 1B,C, Figures S1–S3 and S4A–F, Table S2). As TMFI across different regions showed comparable associations with biomarkers, mortality, and disease outcomes, subsequent analyses focused on total TMFI (hereafter referred to simply as TMFI). The associations of TMFI with health outcomes remained broadly consistent in sensitivity analyses using delayed‐entry designs or excluding participants with any outcome disease at baseline, although some associations were attenuated (Figure S5A–D, Table S2). Given the correlation between TMFI and body mass index (BMI), we further adjusted for BMI, and the associations of TMFI with mortality and disease outcomes remained largely unchanged (Figure S5E). Interestingly, TMFI exhibited significant multiplicative interactions with age and sex for several health outcomes. Overall, the associations between TMFI and outcomes were more pronounced in individuals aged < 60 years and in men (Figure S6, Table S3). In addition, TMFI was significantly positively associated with the prevalence of all systemic diseases (Figure S5F, Table S2). Individuals in the highest TMFI quartile had shorter life expectancy after age 40 than those in the lowest quartile (Figure 1D). For most system‐specific diseases, the early‐onset group exhibited higher average TMFI than the late‐onset group, whereas the disease‐free group showed the lowest values (Figure 1E, Tables S4 and S5).

### 2.2 TMFI Mediates the Influence of Lifestyle Factors on Health Outcomes

**FIGURE 2:**
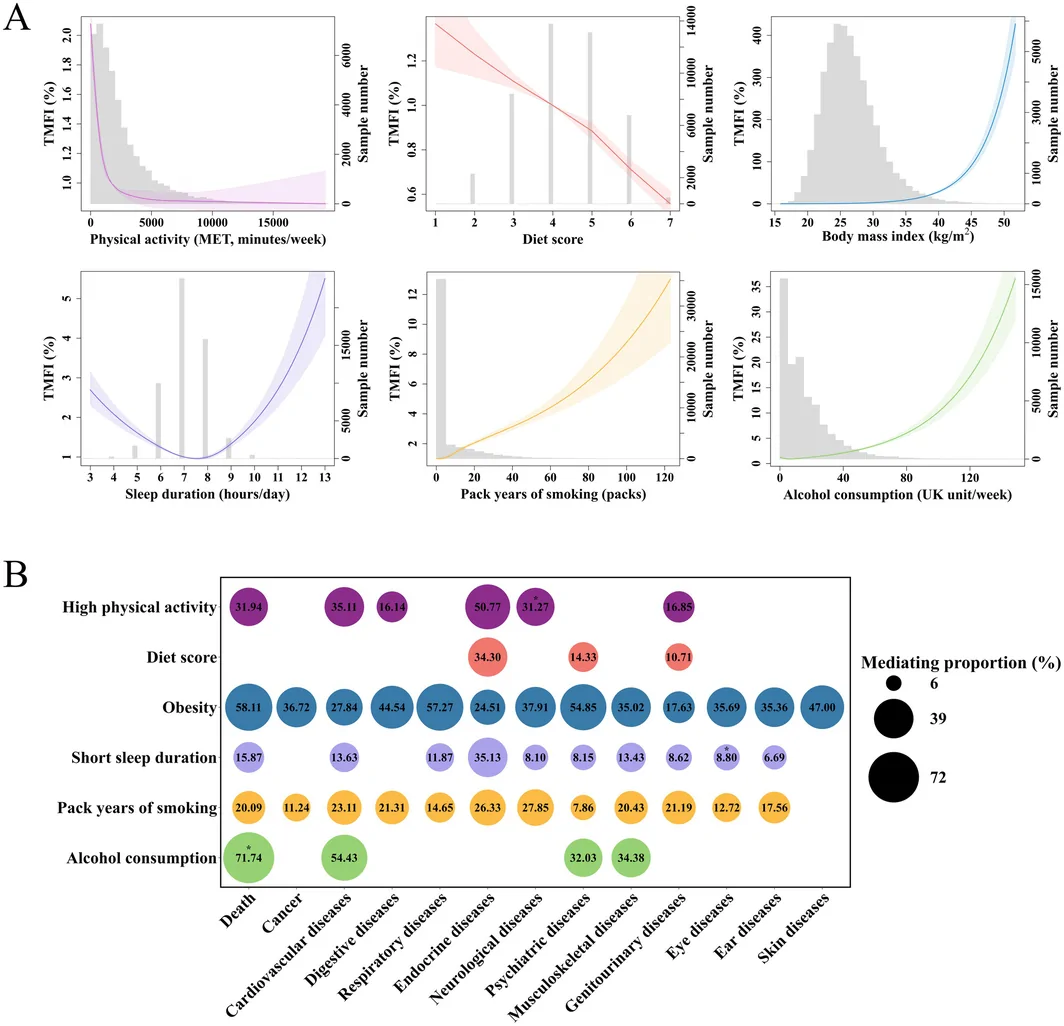
Thigh muscle fat infiltration mediates the effects of lifestyle factors on health outcomes. (A) Restricted cubic spline plots depicting the associations between lifestyle exposures and thigh muscle fat infiltration (TMFI). (B) Bubble plot depicting mediation proportions of TMFI in the associations of modifiable factors with mortality and the incidences of multiple diseases. Bubble size reflects the mediating proportion of TMFI. *Indicates that the association between exposure and outcome was not statistically significant in the mediation analysis, whereas it was significant in the original TMFI dataset. This difference is attributable to bootstrap resampling in the mediation framework, where each iteration generates a resampled dataset, leading to variation in the analytic samples compared with the original TMFI dataset.

To investigate the impact of lifestyle factors on TMFI, we applied restricted cubic splines (RCS) to describe the dose–response relationships between lifestyle factors and TMFI. The analysis revealed that diet quality, alcohol consumption, and smoking exhibited approximately linear associations with TMFI; physical activity and BMI showed L‐shaped and reverse L‐shaped relationships, respectively, whereas sleep duration displayed a U‐shaped association, with the lowest TMFI observed at 7.52 h/day of sleep time (Figure 2A).

Subsequently, we examined the associations of lifestyle factors—including high physical activity, diet quality, obesity, long sleep duration, short sleep duration, smoking, and alcohol consumption—with all‐cause mortality and the incidence of system‐specific diseases (Figure S7, Table S6). For lifestyle factors significantly associated with health outcomes, we further examined the mediating role of TMFI in these associations. The results indicated that TMFI significantly mediated all of these relationships (Figure 2B, Table S7), suggesting that lifestyle factors may influence mortality and system‐specific diseases through TMFI.

### 2.3 Genomic Loci and Mapped Genes Associated With TMFI

**FIGURE 3:**
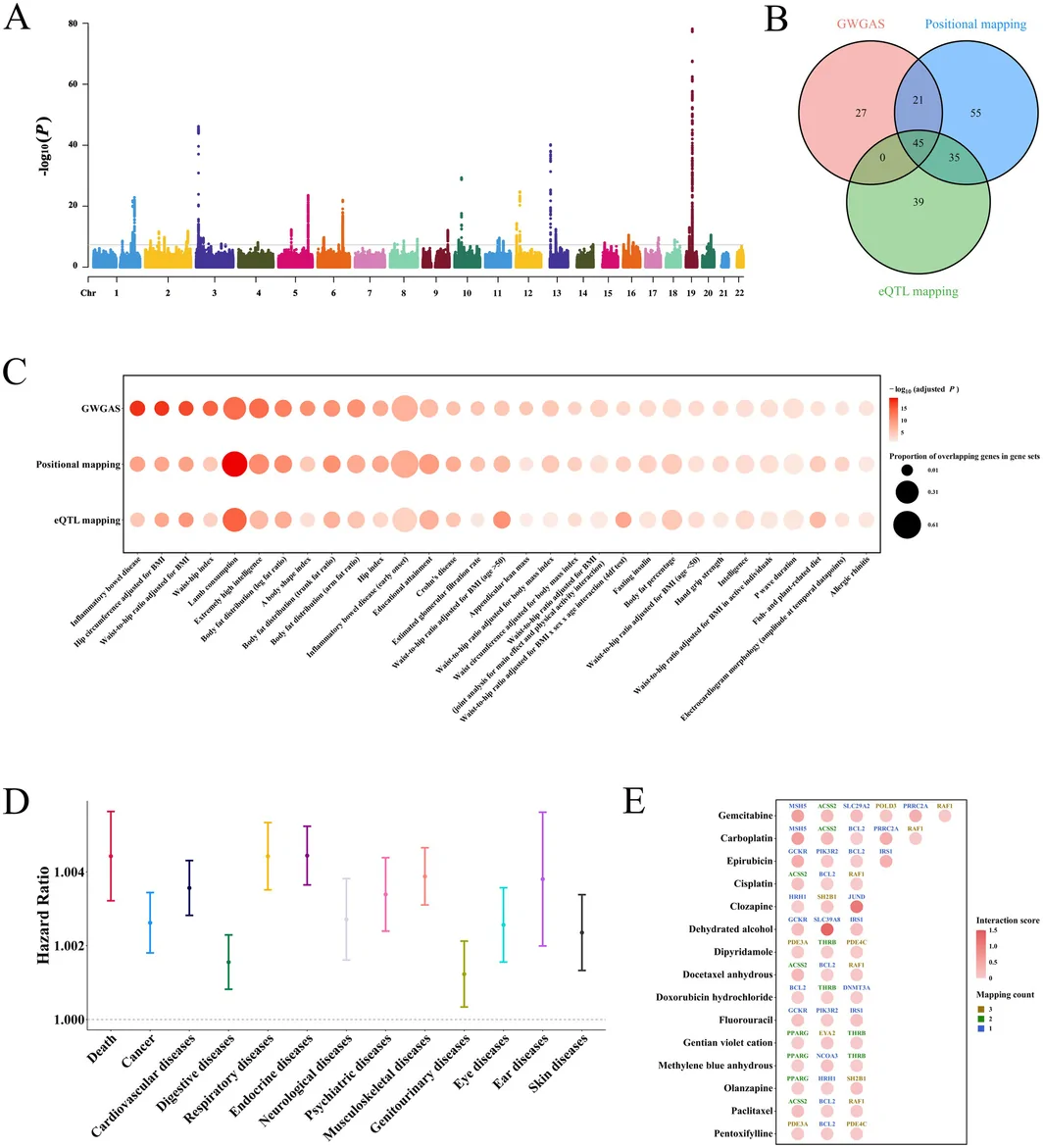
Genetic loci and mapped genes underlying thigh muscle fat infiltration. (A) Manhattan plot depicting the genome‐wide association study (GWAS) for thigh muscle fat infiltration (TMFI). The horizontal line represents the GWAS significance level (*p* = 5 × 10^−8^). (B) Venn diagram depicting the number of genes associated with TMFI revealed by positional mapping, expression quantitative trait locus (eQTL) mapping, and genome‐wide gene‐based association study (GWGAS). (C) Bubble plot depicting the enrichment of mapped genes from three approaches in the GWAS Catalog gene sets. The names of gene sets from the GWAS Catalog are displayed below the corresponding bubbles. Bubble size reflects the proportion of mapped genes within each gene set. Color intensity indicates the false discovery rate (FDR)‐corrected statistical significance of enrichment. Gene sets are ordered by decreasing significance from the GWGAS analysis. (D) Forest plot depicting hazard ratios for associations of the polygenic risk score (PRS) for TMFI with mortality and the incidences of multiple diseases in the non‐GWAS cohort. (E) Dot plot depicting food and drug administration (FDA)‐approved drugs targeting at least three TMFI‐associated genes. Dot color indicates drug‐gene interaction strength. Gene label colors denote the number of supporting approaches (positional mapping, eQTL mapping, and GWGAS) for each gene. The PRS was calculated using the clumping‐and‐thresholding (C + T) method. EQTL, expression quantitative trait locus; GWGAS, genome‐wide gene‐based association study.

Through GWAS, we identified 79 lead single nucleotide polymorphisms (SNPs) significantly associated with TMFI at genome‐wide significance (*p* < 5 × 10^−8^), mapping to 47 genomic loci, most of these loci have not been previously reported in relation to TMFI (Figure 3A, Figure S8A–C, Table S8). To further explore the potential functional genes affected by these risk SNPs, we performed a genome‐wide gene‐based association study (GWGAS) using MAGMA, as well as positional mapping and expression quantitative trait loci (eQTL) mapping via FUMA. These approaches identified 93, 156, and 119 unique genes, respectively, with 45 genes overlapping across all three methods (Figure 3B, Tables S9–S11). Comparison with genes reported in the GWAS catalog revealed that the mapped genes were significantly enriched for traits related to body size, fat distribution, and inflammatory diseases (Figure 3C, Table S13). Gene‐tissue expression analysis based on MAGMA further demonstrated that TMFI‐associated genes were highly enriched in skeletal muscle, adipose visceral omentum, and reproductive tissues (Figure S8D). Subsequently, we constructed a polygenic risk score (PRS) for TMFI based on the GWAS results. Analysis in the non‐GWAS cohort revealed that a higher PRS was significantly associated with increased risks of all‐cause mortality and almost all major system‐specific diseases (Figure 3D, Figure S9, Table S14). We further investigated the interactions between the PRS and lifestyle factors on TMFI. Multiple lifestyle factors exhibited significant multiplicative interactions, such that the adverse effect of unhealthy lifestyles was amplified in individuals with high genetic risk, as well as additive interactions, suggesting a synergistic effect of poor lifestyles and high genetic risk on TMFI (Figure S10, Tables S15 and S16). To evaluate the potential for pharmacological targeting of the identified genes, we queried the Drug‐Gene Interaction Database (DGIdb) for gene‐drug interactions. A total of 58 genes were found to interact with 901 drugs. Notably, 15 drugs targeted three or more of the mapped genes, highlighting potential candidate drugs that may modulate TMFI (Figure 3E, Table S17). Given that DGIdb does not provide directionality of drug effects and that many identified compounds are broad‐spectrum agents with extensive gene target profiles, these findings should be interpreted as hypothesis‐generating.

### 2.4 Cell Types and Pathway Enrichments Associated With TMFI

**FIGURE 4:**
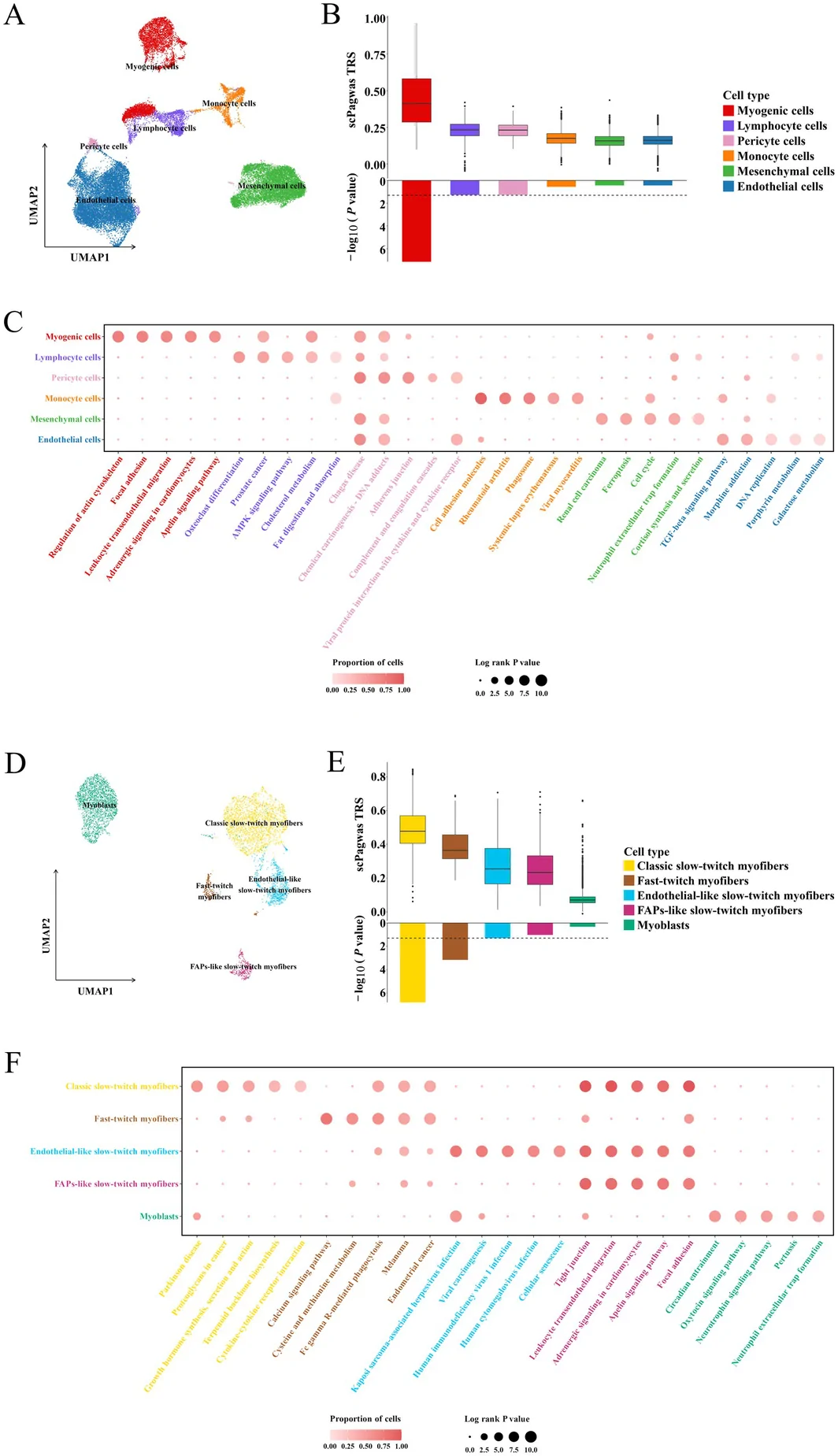
Cell types and enriched pathways associated with thigh muscle fat infiltration. (A) Uniform manifold approximation and projection (UMAP) plot depicting 33,975 single‐cell transcriptomes of thigh muscle cells. (B) Trait‐relevant scoring prioritizes thigh muscle cell types linked to thigh muscle fat infiltration (TMFI). Boxplots depict the distributions of trait‐relevant score (TRS) for six thigh muscle cell types. (C) Bubble plot depicting top‐enriched pathways across cell types of thigh muscle cells. (D) UMAP plot depicting 5527 single‐cell transcriptomes of myogenic cells. (E) Trait‐relevant scoring prioritizes myogenic cell types. Boxplots depict the distributions of TRS for five myogenic cell sub‐types. (F) Bubble plot depicting top‐enriched pathways across cell sub‐types of myogenic cells. In Panels B and E, the upper, middle, and lower lines of the boxes represent the upper quartile, median, and lower quartile of TRS, respectively. Bar height indicates the −log_10_ *p* value for each cell type, and the horizontal dashed line represents the significance threshold (*p* = 0.05). In Panels C and F, each cell type displays the top five pathways with the lowest *p* values, corresponding to text colors. Bubble size represents the log‐ranked *p* value for each pathway, and color intensity indicates the proportion of cells within each cell type genetically influenced by a given pathway. scPagwas, single‐cell pathway‐associated genome‐wide association study; TRS, trait‐relevant score; UMAP, uniform manifold approximation and projection.

To identify the cell types associated with TMFI, we integrated GWAS summary statistics of TMFI with single‐cell transcriptomic data from the vastus lateralis muscle and performed an scPagwas analysis. Based on cell‐type‐specific marker genes, cells from the vastus lateralis were classified into six major types. Among these, only myogenic cells showed significant genetic relevance to TMFI and exhibited the highest trait‐relevant score (TRS) (Figure 4A,B, Figure S11A,B, Table S18). Pathway enrichment analysis revealed cell‐type‐specific pathways associated with TMFI (Figure 4C, Table S19). To further dissect the influence of myogenic cells on TMFI, we clustered them into five distinct cell subpopulations, including classical myoblasts, slow‐twitch myofibers, and fast‐twitch myofibers, as well as slow‐twitch myofibers exhibiting endothelial features (termed endothelial‐like slow‐twitch myofibers) and slow‐twitch myofibers displaying fibro/adipogenic progenitor cells‐like characteristics (termed FAPs‐like slow‐twitch myofibers). scPagwas analysis identified three cell subpopulations as TMFI‐relevant cell populations, with classic slow‐twitch myofibers exhibiting the highest TRS, followed by fast‐twitch myofibers and endothelial‐like slow‐twitch myofibers (Figure 4D,E, Figure S11C,D, Table S20). Pathway enrichment analysis suggested that the subpopulations of myogenic cells may influence TMFI through distinct potential pathways (Figure 4F, Table S21).

### 2.5 The Impact of Multi‐Tissue Gene Expression on TMFI

**FIGURE 5:**
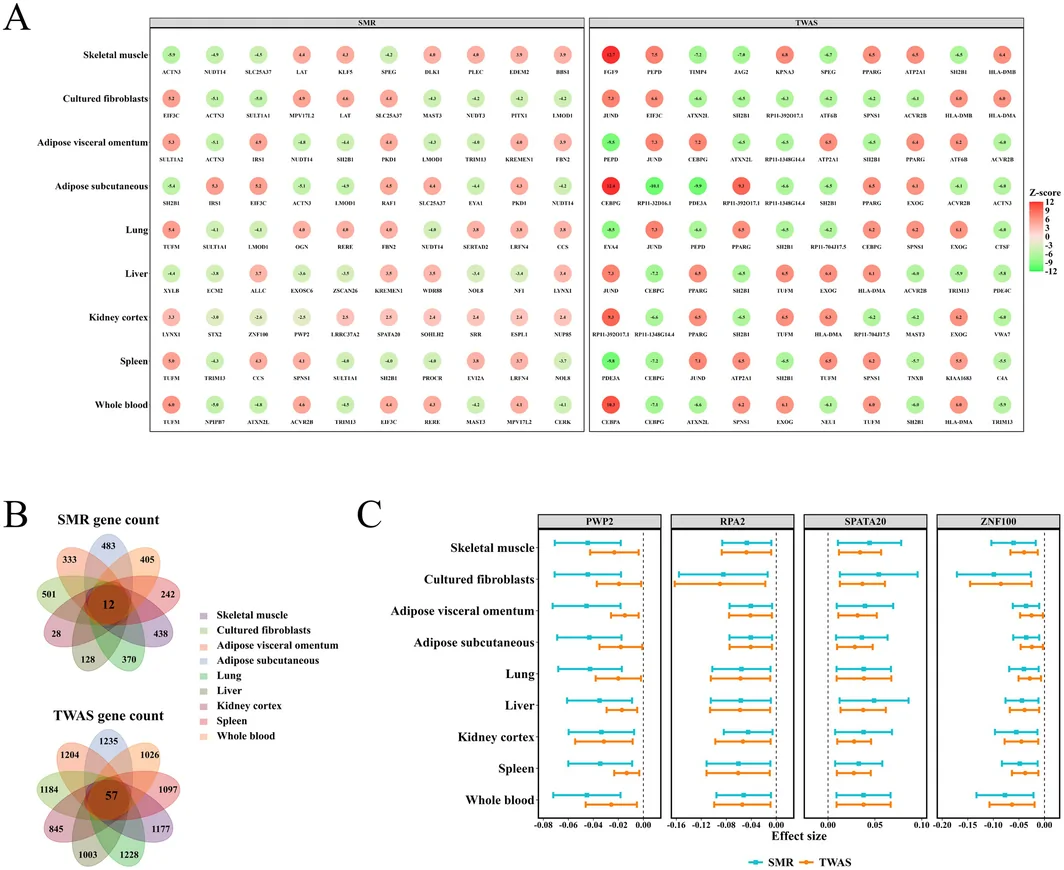
Gene expression across multiple tissues affects thigh muscle fat infiltration. (A) Dot plots depicting the top 10 Thigh Muscle Fat Infiltration (TMFI)‐associated genes with the highest absolute *Z*‐scores from SMR or TWAS analyses across diverse tissues potentially related to the TMFI. The 9 examined tissues potentially related to the TMFI are labeled on the left side of the dots. Dot color indicates the *Z*‐score direction and magnitude. (B) Venn diagrams depicting the number of TMFI‐associated genes identified by TWAS or SMR analyses across diverse tissues potentially related to the TMFI. (C) Forest plots depicting the effect sizes and their 95% confidence intervals for the four genes consistently identified by both SMR and TWAS across tissues potentially related to the TMFI. SMR, summary‐data‐based Mendelian randomization; TWAS, transcriptome‐wide association study.

To examine the effects of gene expression in tissues potentially related to TMFI, we integrated tissue‐specific eQTL data from the GTEx project with TMFI GWAS summary statistics and performed summary‐data‐based Mendelian randomization (SMR) and transcriptome‐wide association study (TWAS) analyses. Both approaches identified numerous genes potentially associated with TMFI across the nine analyzed tissues (Figure 5A, Tables S22 and S23). Specifically, 12 genes detected by SMR and 57 genes detected by TWAS showed associations with TMFI in every analyzed tissue (Figure 5B, Figure S12). The majority of these genes exhibited consistent directions of effect, suggesting cross‐tissue concordance in their regulatory roles. Four genes were concurrently identified by both methods. Among these, SPATA20 was positively associated with TMFI across all the tissues, whereas PWP2, RPA2, and ZNF100 showed negative associations (Figure 5C, Table S24).

### 2.6 Circulating Proteins and Metabolites Associated With TMFI

**FIGURE 6:**
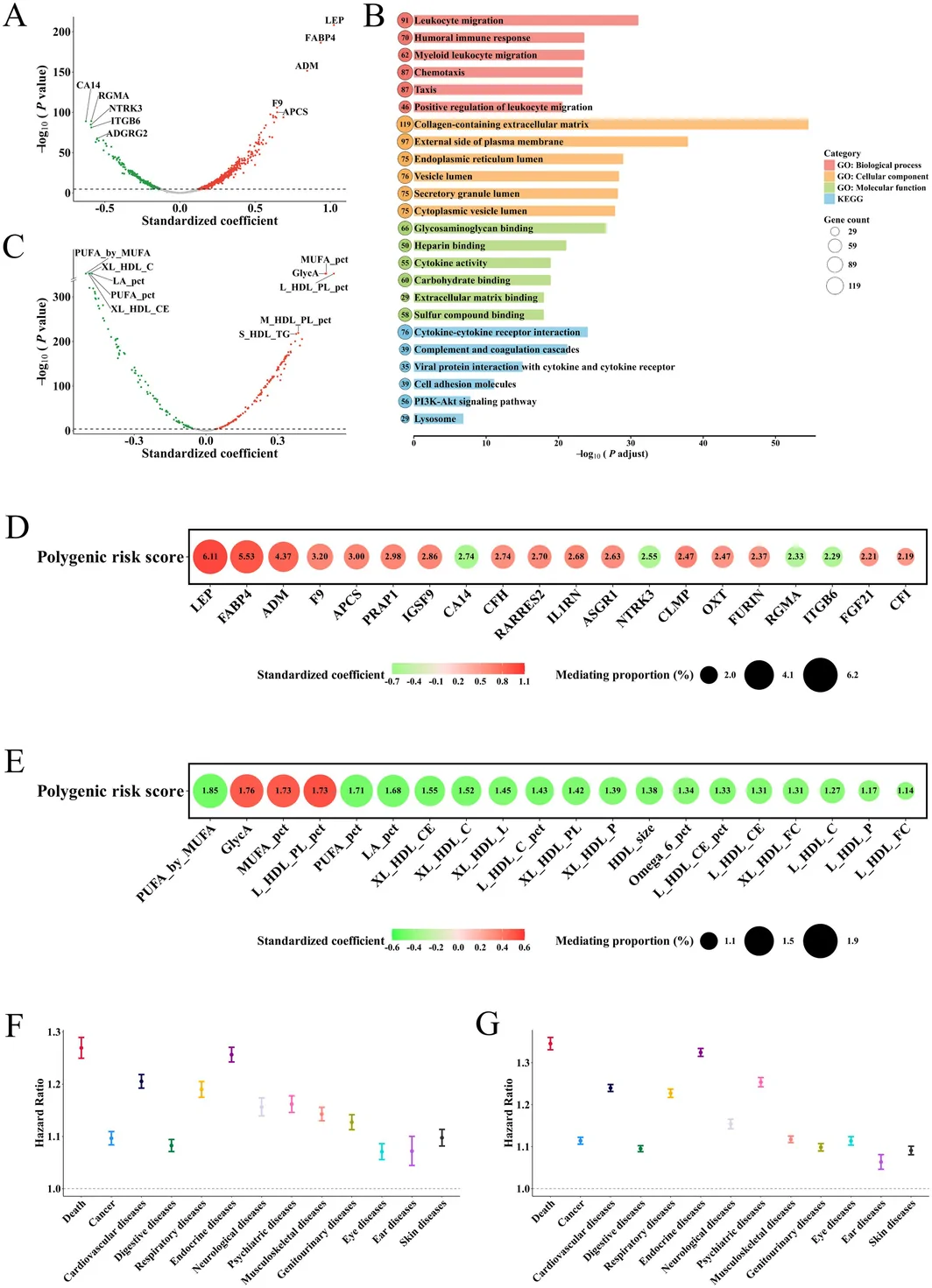
Circulating Proteins and Metabolites Linked to Thigh Muscle Fat Infiltration. (A) Volcano plot depicting associations between 2923 plasma circulating proteins and thigh muscle fat infiltration (TMFI). Horizontal dashed line shows the Bonferroni‐corrected significance threshold (*p* = 0.05/2923). (B) The top significantly enriched GO and KEGG pathways for TMFI‐associated plasma proteins. Bar length represents the statistical significance of enrichment. The size of the bubbles and the numbers indicate the count of enriched genes. (C) Volcano plot depicting associations between 251 plasma metabolites and TMFI. Horizontal dashed line denotes the Bonferroni‐corrected significance threshold (*p* = 0.05/251). (D) Bubble plot depicting plasma circulating proteins significantly mediating the association between polygenic risk score (PRS) and TMFI. The top 20 proteins with the highest mediation proportions are presented. (E) Bubble plot depicting plasma metabolites significantly mediating the association between PRS and TMFI. The top 20 metabolites with the highest mediation proportions are presented. (F) Forest plot depicting hazard ratios for associations of TMFI proteomic signature with mortality and the incidences of multiple diseases. (G) Forest plot depicting hazard ratios for associations of TMFI metabolomic signature with mortality and the incidences of multiple diseases. In volcano plots, red dots indicate significant positive associations, green dots represent significant negative associations, gray dots show no significant associations, the *y*‐axis displays the −log_10_ *p* value for each protein or metabolite, while the *x*‐axis displays the standardized coefficients of their associations with TMFI. In bubble plots of mediation analysis, bubble color depicts the standardized coefficient from linear regression analysis of the effect of the mediator on TMFI, while bubble size reflects the mediating proportion of the mediator. The PRS was calculated using the clumping‐and‐thresholding (C + T) method. GO, Gene Ontology; KEGG, Kyoto Encyclopedia of Genes and Genomes.

To explore the potential influence of circulating biomarkers on TMFI, we examined the associations of circulating proteins and metabolites with TMFI in participants with proteomic and metabolomic profiling, respectively. Among the 2923 proteins analyzed, 977 were significantly associated with TMFI (*p* < 0.05/2923). The top 10 proteins showing the strongest positive associations with TMFI were LEP, FABP4, ADM, CLMP, APCS, F9, IGSF9, PRAP1, IL1RN, and RARRES2, whereas the top 10 showing the strongest negative associations were CA14, NTRK3, RGMA, ITGB6, ART3, IGDCC4, ADGRG2, MXRA8, ENPP6, and PON3 (Figure 6A, Table S25). Tissue localization analysis of these proteins based on the GTEx and HPA databases revealed that immune tissues, liver, and brain were consistently the top sources (Figure S13), suggesting that these tissues may play important roles in TMFI pathogenesis. Pathway enrichment analysis showed that the proteins were primarily involved in leukocyte migration and chemotaxis, humoral immune response, extracellular matrix organization, signal transduction regulation, PI3K‐Akt signaling pathway, and lysosomal function (Figure 6B, Tables S26 and S27). Meanwhile, among the 251 metabolites analyzed, 211 were significantly associated with TMFI (*p* < 0.05/251) (Figure 6C, Table S28). Notably, the metabolites most strongly associated with TMFI were largely related to lipid metabolism, suggesting its key role in TMFI. We further investigated whether circulating biomarkers mediate the associations between lifestyle factors or genetic risk and TMFI, and identified multiple proteins and metabolites with significant mediating effects (Figure 6D,E, Figures S14 and S15, Tables S29 and S30). Tissue localization and pathway enrichment analyses of these mediating proteins revealed distinct tissue and pathway enrichment patterns depending on the exposure (Figure S16, Tables S31 and S32). Finally, we leveraged deep learning to develop proteomic and metabolomic signature scores for TMFI, achieving test‐set Pearson correlations with measured TMFI of 0.7059 and 0.4909, respectively (Table S33). Both scores were strongly associated with mortality and the incidence of system‐specific diseases (Figure 6F,G, Table S34), indicating that circulating biomarkers can reliably capture biological signals of TMFI and reflect its widespread impact on systemic health.

## 3 Discussion

Based on a large population cohort and multi‐omics approach, this study is the first to systematically characterize the health implications, determinants, and molecular mechanisms of TMFI. We found that TMFI was positively associated with all‐cause mortality and multiple system‐specific diseases, exhibiting a clear dose–response relationship. Further analyses suggested that TMFI may mediate the effects of unhealthy lifestyles on clinical outcomes. Through a GWAS of TMFI, we identified multiple genetic risk loci, with mapped genes significantly enriched for traits related to body morphology, fat distribution, and inflammatory diseases, and preferentially expressed in tissues such as skeletal muscle, adipose visceral omentum, and the reproductive system. Leveraging single‐cell transcriptomic data from the vastus lateralis muscle, we demonstrated that classic slow‐twitch myofibers, fast‐twitch myofibers, and endothelial‐like slow‐twitch myofibers within the myogenic cells were associated with TMFI. Integrating SMR and TWAS analyses, we detected several genes whose expression levels were robustly linked to TMFI across multiple tissues. Proteomic and metabolomic profiling revealed a large set of TMFI‐associated circulating biomarkers, and deep learning‐derived proteomic and metabolomic signatures of TMFI were both associated with mortality and the incidence of all major systemic diseases. Collectively, our study highlights the biological significance of TMFI in human health, elucidates its potential mechanisms from a multi‐omics perspective, and identifies promising targets to inform precision prevention and intervention.

Previous studies have shown that TMFI is associated with all‐cause mortality, heart failure, nonalcoholic fatty liver disease, and adverse cancer outcomes (Linge et al. 2021; Huynh et al. 2022). However, most existing studies focused on single diseases or specific clinical outcomes, lacking a comprehensive evaluation of TMFI's health implications. The present study systematically examined the associations of TMFI with all‐cause and cause‐specific mortality, as well as a broad spectrum of diseases, providing evidence for its widespread impact on human health. Building on this, and given that unhealthy lifestyles have been reported to affect TMFI (Goodpaster et al. 2008; Ogawa et al. 2021; Prokopidis et al. 2025; Santanasto et al. 2011), we further investigated the mediating role of TMFI in the associations between lifestyle factors and health outcomes. As expected, lifestyle factors may influence health outcomes in part through their effects on TMFI, underscoring the importance of maintaining a healthy lifestyle to reduce TMFI and preserve overall health.

To date, GWAS on muscle fat infiltration remain limited, and its genetic architecture has not been fully explored. We performed a comprehensive GWAS of TMFI. By contrast with previous studies (Tan et al. 2026; van der Meer et al. 2022), we incorporated TMFI data from four anatomical sites and conducted analyses in a large‐scale sample, facilitating the discovery of novel TMFI‐associated risk loci and genomic regions. GWAS‐based gene mapping showed that the mapped genes were significantly enriched for traits related to body composition, fat distribution, and inflammatory diseases. Body composition traits, such as BMI, have been shown to correlate positively with TMFI (Joseph et al. 2025). Similarly, inflammatory diseases are often accompanied by elevated TMFI and impaired muscle function, as inflammatory mediators can promote lipid deposition within skeletal muscle (Avesani et al. 2023; Addison et al. 2014). Gene–tissue expression analyses showed that the mapped genes were enriched in skeletal muscle, adipose visceral omentum, and reproductive tissues, which is biologically plausible. Skeletal muscle is central to whole‐body glucose and fatty acid utilization (Merz and Thurmond 2020; Jensen 2002), and damaged muscle fibers or reduced metabolic activity lead to lipid accumulation (Gorgey and Dudley 2007). Excess visceral fat typically coexists with systemic inflammation, insulin resistance, and dyslipidemia, all of which can contribute to muscle fat infiltration (Wang et al. 2024; Chait and den Hartigh 2020). Sex hormone levels also influence TMFI, with estrogen decline (e.g., postmenopause) and testosterone changes linked to fat redistribution and increased intramuscular fat (Tiidus 2011; Ronkainen et al. 2009; Hamrick et al. 2016; Kelly and Jones 2013). Some GWAS‐mapped genes identified in our study, such as *PEPD*, have also been implicated in previous TMFI‐related GWAS findings (Tan et al. 2026; van der Meer et al. 2022). Notably, *PEPD* expression and activity are reduced in adipose tissue under obese conditions and have been linked to tissue fibrosis and insulin resistance (Pellegrinelli et al. 2022). However, its specific role in TMFI has not yet been elucidated and warrants further investigation. Importantly, more than half of the mapped genes, such as *ATG7* and *XYLB*, were implicated in TMFI for the first time. *ATG7* is a core autophagy gene, and autophagy is known to influence lipid droplet metabolism and tissue fat deposition. A study has demonstrated that ATG7 plays an essential role in adipogenesis and adipose tissue homeostasis (Zhang et al. 2009). *XYLB* encodes xylulokinase, which produces xylulose‐5‐phosphate to activate lipogenic pathways and promote carbohydrate‐induced lipid synthesis, suggesting that *XYLB* may influence TMFI through glucose‐driven lipogenesis (Bunker et al. 2013). In summary, this study identified a set of novel genetic risk loci associated with TMFI, thereby enhancing our understanding of its underlying genetic mechanisms.

We constructed a PRS to capture individual genetic susceptibility to TMFI and found that a higher PRS was significantly associated with increased risks of mortality and multiple system‐specific diseases. To our knowledge, this is the first systematic investigation of the relationship between TMFI genetic risk and health outcomes. Our findings suggest that genetic susceptibility to TMFI may exert broad effects on overall health. Additionally, we examined the interaction effects between the PRS and lifestyle factors on TMFI, revealing both multiplicative and additive interactions between genetic risk and various lifestyle factors. These results indicate that individuals with higher genetic risk may be particularly susceptible to adverse lifestyle factors, highlighting the importance of healthy lifestyle behaviors in mitigating TMFI progression.

Drug‐target analysis of GWAS‐mapped genes identified 15 drugs that each interact with at least three genes; however, whether these drugs increase or decrease TMFI should be addressed in future studies. It is noteworthy that several drugs, such as Dipyridamole and Pentoxifylline, have been demonstrated to improve muscle quality by enhancing microcirculation, exerting anti‐inflammatory effects, and promoting muscle regeneration (Marco‐Bonilla et al. 2023; Arcaro et al. 2018). Similarly, Methylene Blue has been reported to enhance mitochondrial function, suppress oxidative stress, and exert anti‐inflammatory effects (Atamna et al. 2008; Ahn et al. 2017), suggesting a potential muscle‐protective role. We also showed that multiple anticancer chemotherapeutic agents, including Cisplatin, Carboplatin, Paclitaxel, Docetaxel, Doxorubicin, Epirubicin, Gemcitabine, and Fluorouracil, are potential modulators of TMFI. Most of these agents have been reported to reduce skeletal muscle mass, decrease muscle radiodensity, and increase intramuscular fat (Conte et al. 2020; Huang et al. 2025; Mallard et al. 2021), highlighting the need to consider their potential adverse effects on muscle during clinical use in cancer patients, especially given the association between TMFI and poor cancer prognosis (Aleixo et al. 2020).

Our results suggest that myogenic cells may play a pivotal role in TMFI. They can secrete a variety of signaling molecules that modulate the interstitial microenvironment and inflammatory responses, potentially regulating intramuscular fat deposition (Hamrick et al. 2016; Yin et al. 2013). Upon further subclassification of myogenic cells, we found that classic myofibers (including slow‐twitch and fast‐twitch myofibers) and endothelial‐like slow‐twitch myofibers (exhibiting both slow‐twitch and endothelial molecular features) were significantly associated with TMFI. Myofibers, the fundamental structural and functional units of skeletal muscle, are responsible for contraction, postural maintenance, and energy metabolism, and are categorized into slow‐twitch and fast‐twitch types (Frontera and Ochala 2015). Damage or atrophy of myofibers can trigger aberrant differentiation of fibro/adipogenic progenitor cells into adipocytes, thereby contributing to fat infiltration (Villalobos et al. 2025). Additionally, a reduction in slow‐twitch myofibers or an increase in fast‐twitch myofibers is recognized as a key feature of muscle fat infiltration. Such shifts diminish oxidative metabolic capacity and impair lipid clearance efficiency, ultimately facilitating fat infiltration (Lloyd et al. 2023; Umek et al. 2021; Li et al. 2025). Notably, the absence of significant associations for certain cell types does not imply they are biologically irrelevant to TMFI; rather, it may reflect their limited contribution to the genetic architecture of TMFI.

SMR and TWAS analyses in relevant tissues identified numerous genes potentially influencing TMFI, suggesting involvement of complex regulatory networks. Of note, four genes exhibited significant and directionally consistent associations with TMFI across all nine tissues examined in both SMR and TWAS analyses. Specifically, SPATA20 showed a positive association with TMFI, whereas PWP2, RPA2, and ZNF100 showed negative associations. PWP2, a conserved gene with RNA‐binding activity, is essential for small ribosomal subunit formation and precursor rRNA processing (Boissier et al. 2017). RPA2, a core subunit of the replication protein A complex, stabilizes single‐stranded DNA during replication and repair, participating in DNA replication, recombination, and the DNA damage response (Dueva and Iliakis 2020). As a zinc finger protein, ZNF100 likely regulates gene transcription through DNA‐binding zinc finger domains (Streicher et al. 2021). Considering their fundamental roles in cellular processes, these genes may influence TMFI across different tissues through similar mechanisms, although the specifics remain unclear. Existing studies have primarily focused on the role of SPATA20 in spermatozoa (Martinez et al. 2023; Wang et al. 2023), whereas a few studies have reported that it promotes oxidative stress responses (Jarvis et al. 2020), which may accelerate muscle fat infiltration (Chen et al. 2023). Additionally, SPATA20 expression increases during adipocyte differentiation, and its variants are associated with arm fat mass (Neville et al. 2019). Altogether, these genes may represent targets for modulating TMFI.

In this study, we investigated the associations between circulating biomarkers and TMFI. Among the proteins most strongly associated with TMFI, leptin showed a positive association. Leptin is a peptide hormone secreted by white adipose tissue (Martínez‐Sánchez 2020) that exerts central regulatory functions primarily through the hypothalamus (Sahu 2003). In addition, leptin promotes adipogenic and lipogenic factor expression and lipid‐droplet formation in preadipocytes in vitro (Martínez‐Sánchez 2020), and induces IL‐6‐mediated muscle inflammation in aged rats (Tazawa et al. 2019). Observational studies link higher plasma leptin levels to greater muscle fat infiltration and weaker muscle strength (Zoico et al. 2010; Kohara et al. 2011), whereas some reports suggest that leptin may instead suppress muscle fat infiltration (Hamrick et al. 2016; Yu et al. 2024). This may imply a dual role for leptin in muscle, and its precise mechanisms warrant further investigation. FABP4 was also positively associated with TMFI. This aligns with previous work showing that tendon rupture in mice increases FABP4 expression, leading to fat accumulation, whereas FABP4 inhibitors attenuate this effect (Lee et al. 2017). Midregional pro‐ADM (a stable fragment reflecting ADM levels) has been reported to positively correlate with TMFI, implicating ADM in intramuscular fat deposition (Akasaka et al. 2025). We similarly observed a positive association between ADM and TMFI. By contrast, NTRK3 was found to be negatively associated with TMFI. Previous research has shown that NTRK3 activation promotes lipolysis and blocks adipocyte precursor differentiation, suggesting a protective effect against TMFI (Bové et al. 2021). RGMA was also negatively associated with TMFI. Although RGMA was initially identified for its role in neuronal development, it has recently been reported to be expressed in skeletal muscle (Copola et al. 2022). RGMA treatment suppresses the expression of atrophy‐related genes, while overexpression of RGMA in myogenic cells enhances nuclei accretion, a process crucial for muscle regeneration (Copola et al. 2022). Tissue‐localization analyses indicated that immune tissues, the liver, and the brain are the major sources of TMFI‐associated circulating proteins, a biologically coherent finding. Immune activation releases pro‐inflammatory cytokines (e.g., IL‐6, TNF‐α) that promote muscle wasting, insulin resistance, and mitochondrial dysfunction, all of which can exacerbate fat infiltration (Bian et al. 2017). Hepatic steatosis induces systemic inflammation and may further drive TMFI (De Munck et al. 2021). Central nervous system disorders can increase gut permeability, which leads to chronic systemic inflammation and insulin resistance, ultimately promoting muscle fat infiltration (Yin et al. 2022). These lines of evidence, together with our protein localization results, indicate that immune tissues, the liver, and the brain are key contributors to TMFI. Furthermore, enrichment analysis of TMFI‐associated circulating proteins implicated multiple biological processes, including leukocyte migration and chemotaxis, humoral immune responses, extracellular matrix assembly, regulation of signal transduction, and lysosomal function, highlighting the complex interplay of diverse biological mechanisms underlying TMFI.

In the analysis of circulating metabolites, the majority of metabolites remained significantly associated with TMFI after multiple testing correction, indicating a close relationship between metabolic alterations and TMFI. Lower cholesteryl ester content within HDL particles generally reflects impaired HDL maturation and transport capacity, leading to reduced lipid clearance in peripheral tissues (Marques et al. 2018). Very large HDL particles represent the most mature and functionally active HDL subfractions, and their cholesteryl ester content (XL_HDL_CE) serves as a sensitive marker of HDL functional integrity. In our study, XL_HDL_CE was negatively associated with TMFI, suggesting that lower levels of XL_HDL_CE may contribute to TMFI through reduced lipid clearance. Glycoprotein Acetyls (GlycA) is a well‐established marker of systemic inflammation (Connelly et al. 2017). We found a strong positive association between GlycA and TMFI, supporting a role of inflammation in the biology of TMFI. Linoleic acid, an essential polyunsaturated fatty acid (PUFA) obtained from the diet, is important for maintaining normal physiological functions (Kapoor et al. 2021). Linoleic Acid to Total Fatty Acids percentage (LA_pct) and PUFAs to Total Fatty Acids percentage (PUFA_pct) were found to be negatively associated with TMFI in this study, suggesting that maintaining a healthy diet may be protective against TMFI.

Moreover, multiple proteins and metabolites were identified as mediators of the effects of lifestyle factors and genetic risk on TMFI. Tissue‐localization and pathway enrichment analyses revealed distinct tissue origins and biological pathways for mediator proteins corresponding to different exposures, indicating that these factors may influence TMFI through heterogeneous mechanisms. To explore whether circulating biomarkers could serve as proxies for TMFI, we separately constructed proteomic and metabolomic signatures of TMFI. Both signatures exhibited associations with health outcomes similar to those of TMFI itself. These findings suggest that proteomic and metabolomic signatures not only capture TMFI‐related biological processes but also reflect its broader impact on health.

Several limitations of this study should be acknowledged. First, because UKB participants are mainly middle‐aged and older adults, this may limit the generalizability of our findings to younger or more diverse populations, and studies including broader age groups are warranted. Second, the cohort consists largely of individuals of European ancestry who are generally healthier and more socioeconomically advantaged (Fry et al. 2017), so caution is needed when extending our findings to populations with different demographic or health profiles. Third, because of data limitations, this study analyzed TMFI at only a single time point and could not examine its longitudinal trajectories. Fourth, plasma metabolomic and proteomic data were available for only a subset of individuals, and the assays captured only a fraction of circulating proteins and metabolites. Studies with larger sample sizes and more comprehensive biomarker profiling may reveal additional TMFI‐related proteins and metabolites, which could further clarify the biological mechanisms underlying TMFI. Fifth, this study only explored potential determinants of TMFI and did not investigate the underlying mechanisms through which TMFI affects overall health, which requires further exploration in future research.

In summary, this study suggests a potential link between TMFI and health outcomes across multiple organ systems. Moreover, we identified TMFI‐associated lifestyle factors, genetic characteristics, relevant cell types, and potential molecular mechanisms. These findings enhance our understanding of TMFI and may offer novel insights for its prevention and intervention.

## 4 Methods

### 4.1 Study Population

All participant information used in this study was obtained from the UKB, a comprehensive biomedical research resource that originally enrolled more than 500,000 individuals aged 37–73 years. Over years of follow‐up, the UKB has accumulated an extensive array of phenotypic and genetic information, including demographic data, standardized clinical assessments, biological specimens, and a wide range of imaging modalities (Sudlow et al. 2015). Ethical approval for UKB was issued by the National Information Governance Board for Health and Social Care and the NHS North West Multicenter Research Ethics Committee (11/NW/0382). Written informed consent was obtained from all participants at the time of recruitment. According to the approved governance procedures, no additional ethics review was required for researchers utilizing UKB data.

### 4.2 Data Collection

This study utilized nondiagnostic information from three consecutive UKB assessment waves: the baseline visit (V0), the imaging assessment (V2), and the first repeat imaging visit (V3). At V0, a wide range of variables was used in the analyses, including age, sex, ethnicity, education, reaction time, forced vital capacity (FVC), forced expiratory volume in 1‐s (FEV1), heel bone mineral density (BMD), left hand grip strength (HGS), right HGS, weight change compared with 1 year ago, frequency of tiredness/lethargy in last 2 weeks, usual walking pace (UWP), types of physical activity in last 4 weeks, frequency of light DIY in last 4 weeks, telomere length, neutrophil count, lymphocyte count, monocyte count, platelet count, C‐reactive protein, genetic data, quantitative data of plasma proteins and metabolites, BMI, physical activity (metabolic equivalent task (MET) minutes per week), pack years of smoking, sleep duration, dietary intake, and alcohol consumption. Grip strength was categorized as weaker or stronger based on the lower or higher value between the two hands. Following previous research (Jiang et al. 2023), frailty severity was quantified using eight indicators—left and right grip strength, BMI, weight change relative to 1 year earlier, frequency of tiredness/lethargy in last 2 weeks, UWP, types of physical activity in last 4 weeks, and frequency of light DIY in last 4 weeks—yielding a total score ranging from 0 to 5. The Systemic Immune‐Inflammation Index (SII) was computed as neutrophil count multiplied by platelet count and divided by lymphocyte count. For the V2 assessment, we extracted and utilized MRI data for eight variables, including left anterior thigh fat‐free muscle volume, right anterior thigh fat‐free muscle volume, left posterior thigh fat‐free muscle volume, right posterior thigh fat‐free muscle volume, left anterior TMFI, right anterior TMFI, left posterior TMFI, and right posterior TMFI. TMFI and thigh fat‐free muscle volume were derived from neck‐to‐knee Dixon MRI scans from the UKB imaging cohort. Image processing was performed using an automated AMRA body composition analysis pipeline, including image calibration, fusion of image stacks, image segmentation, and quantification of fat and muscle volumes (West et al. 2016). Image‐level quality control was conducted to assess image analysability and to identify technical issues such as water–fat swaps, imaging artifacts, and incomplete anatomical coverage (Linge et al. 2018). Scans that did not meet predefined quality criteria were excluded prior to generation of the final imaging‐derived phenotypes (Linge et al. 2018). The calculation of total TMFI was as follows: the TMFI of each of the four thigh regions was multiplied by the fat‐free muscle volume of the corresponding region, then the products were summed and divided by the total fat‐free muscle volume of the four regions. The date of attending the assessment center was also collected at the V2. Participant age at V2 was derived from reported birth year and month and the V2 attendance date, assigning the first day of the birth month as the date of birth. Age in decimal years at V2 was computed as: (assessment date − assigned birth date) in days/365.25 (Mutz et al. 2024). During V3, information regarding paternal and maternal ages at death was collected.

### 4.3 Diagnosis of Health Outcomes

System‐specific diseases in this study were identified using diagnostic categories from the International Statistical Classification of Diseases and Related Health Problems, 10th Revision (ICD‐10). The included system‐specific diseases comprised cancers (C00‐C97, D00‐D48), cardiovascular diseases (I00‐I99), digestive diseases (K00‐K93), respiratory diseases (J00‐J99), endocrine diseases (E00‐E90), neurological diseases (G00‐G99), psychiatric diseases (F00‐F99), musculoskeletal diseases (M00‐M99), genitourinary diseases (N00‐N99), eye diseases (H00‐H59), ear diseases (H60‐H95), and skin diseases (L00‐L99). To enhance diagnostic precision, the Classification of Interventions and Procedures, version 4 (OPCS‐4), was additionally used to supplement diagnoses for 10 of these system‐specific diseases, including cardiovascular diseases (K01‐K74, L01‐L99), digestive diseases (G01‐G82, H01‐H71, J01‐J77), respiratory diseases (E01‐E98), endocrine diseases (B01‐B41), neurological diseases (A01‐A82), musculoskeletal diseases (W01‐W99, V01‐V70), genitourinary diseases (M01‐M86, N01‐N35), eye diseases (C01‐C91), ear diseases (D01‐D28), and skin diseases (S01‐S70). This study evaluated six categories of mortality outcomes: all‐cause mortality, as well as deaths attributable to cancer, cardiovascular disease, digestive disease, respiratory disease, and endocrine disease. Information on death dates and underlying causes for all UKB participants was sourced from National Health Service (NHS) records.

### 4.4 Lifestyle‐Related Factors

This study incorporated several lifestyle‐related variables, including physical activity, diet score, BMI, sleep duration, pack‐years of smoking, and alcohol consumption. Physical activity is defined as the total MET minutes per week for all activity including walking, moderate and vigorous activity. The diet score was constructed based on seven dietary components from the UKB Food Frequency Questionnaire, with each component scored dichotomously. Scores ranged from 0 to 7, with one point assigned when the intake met predefined thresholds and zero otherwise. The specific cutoffs were: fruit intake ≥ 3 servings/day, vegetable intake ≥ 3 servings/day, fish intake ≥ 2 servings/week, whole grain intake ≥ 3 servings/day, refined grain intake ≤ 1.5 servings/day, processed meat intake ≤ 1 serving/week, and unprocessed red meat intake ≤ 1.5 servings/week (Lourida et al. 2019). Alcohol consumption was quantified in UK units to enhance international comparison, with one UK unit corresponding to approximately 8 g of pure ethanol (Topiwala et al. 2022). Conversion factors were as follows: one glass of red/white wine or champagne = 1.7 units; one glass of fortified wine = 1.2 units; one pint of beer/cider = 2.4 units; one measure of spirits/liqueurs = 1 unit; and one glass of other alcoholic beverages (e.g., alcopops) = 1.2 units (Topiwala et al. 2022). The total weekly alcohol units were derived by adding the units consumed across all beverage categories. For participants with only monthly intake information, weekly consumption was estimated by dividing the reported monthly units by 4.3. Pack‐years of smoking for ever‐smokers were derived from their smoking history using the formula: (cigarettes per day/20) × (age at smoking cessation − age at initiation). For individuals who had abstained from smoking for more than 6 months, the formula is: (cigarettes per day/20) × (age at cessation − age at initiation −0.5). Participants who initiated and discontinued smoking before age 16 were coded as not available (NA). For those who began before age 16 but quit after, the starting age was set to 16. Participants reporting identical ages for smoking initiation and cessation and having quit for more than 6 months were assigned a pack‐year value of zero. Lifestyle factors were further categorized for analysis. Physical activity, diet score, alcohol consumption, and pack‐years of smoking were dichotomized into low and high groups using median values. BMI was classified as non‐obesity (< 30 kg/m^2^) or obesity (≥ 30 kg/m^2^). Sleep duration was grouped into short (< 6 h/day), normal (≥ 6 to < 8 h/day), and long (≥ 8 h/day).

### 4.5 Olink Proteomics and NMR Metabolomics Data

The UKB Pharma Proteomics Project (UKB‐PPP) quantified 2923 circulating protein biomarkers in 54,306 plasma samples using the Olink Explore 1536 platform based on proximity extension assay (PEA) technology (Goeminne et al. 2025). Independently, the UKB Metabolomics Project examined around 280,000 plasma samples using a high‐throughput nuclear magnetic resonance (NMR) platform (Bruker AVANCE IIIHD) to quantify 251 metabolic biomarkers (Julkunen et al. 2023). Both proteomic and metabolomic measurements were obtained from the V0 of the UKB.

### 4.6 Genome‐Wide Association Study of TMFI

A GWAS of TMFI was performed using genetic data from 46,347 participants of European ancestry who underwent TMFI MRI scans at the V2 in the UKB, using genotype data aligned to the GRCh37 reference genome. Prior to analysis, quality control was applied using PLINK2, excluding SNPs with a missing call rate > 0.05, minor allele frequency (MAF) < 0.001, Hardy–Weinberg equilibrium (HWE) *p* value < 1 × 10^−6^, and imputation information score < 0.8. The GWAS was conducted across all autosomal chromosomes employing a linear mixed model (LMM) framework implemented via the fastGWA‐mlm method in GCTA software (v1.94.1). The model was adjusted for age, genetic sex, education, genotyping batch, and the first 20 genetic principal components.

### 4.7 FUMA‐Based Post‐GWAS Analysis

Gene mapping was performed using the SNP2GENE module in FUMA (version 1.8.0, https://fuma.ctglab.nl/) based on GWAS summary statistics. In this module, Lead SNPs were defined as GWAS‐significant variants (*p* < 5 × 10^−8^) with Linkage Disequilibrium (LD) *r* ^2^ < 0.1, and independent significant SNPs as GWAS‐significant variants with LD *r* ^2^ < 0.6, based on ancestry‐matched 1000 Genomes Project Phase 3 (KGP3) reference genotypes. Independent significant SNPs were mapped to genes through positional mapping (10‐kb window) and eQTL mapping using Genotype‐Tissue Expression (GTEx) v8 data from subcutaneous adipose, visceral omentum adipose, and skeletal muscle tissues (Nievergelt et al. 2024). A genome‐wide gene‐based association study (GWGAS) was performed using the multi‐marker analysis of genomic annotation (MAGMA, version 1.10) implemented in FUMA. Gene‐based association analysis was performed in MAGMA using stringent intragenic mapping, whereby SNPs were assigned exclusively to genes in which they were physically located within the transcriptional boundaries (window size = 0 kb). Multiple testing was controlled using the Bonferroni correction. Using the SNP2GENE module, candidate SNPs (LD *r* ^2^ ≥ 0.6 of any independent significant SNPs and GWAS *p* values < 0.05) were annotated for functional categories via ANNOVAR. Enrichment for each category was calculated as the ratio of annotated candidate SNPs to annotated SNPs in the reference panel, and a two‐sided Fisher's exact test was conducted for statistical assessment. To investigate tissue‐specific enrichment of TMFI‐associated genes, MAGMA‐based gene‐tissue expression analysis was performed using expression profiles from 54 tissues in the GTEx v8 database. Statistical significance was defined by a one‐sided *t*‐test with *p* < 0.05. Additionally, gene set enrichment analysis was performed using the GENE2FUNC module in FUMA to assess whether the mapped genes were significantly overrepresented within predefined gene sets from the GWAS Catalog and the Molecular Signatures Database (MsigDB) c1 (Xu et al. 2025). The hypergeometric test was used to assess enrichment across functionally annotated gene sets, including canonical pathways and Gene Ontology (GO) biological processes. *p* values were adjusted for multiple testing using the Benjamini–Hochberg (BH) procedure, with a significance threshold of 0.05. Only gene sets containing at least two overlapping genes were reported to ensure the robustness of the enrichment results.

### 4.8 Polygenic Risk Score for TMFI

The PRS for TMFI was derived using TMFI GWAS summary statistics and subsequently evaluated for its associations with mortality and the incidence of multiple diseases in an independent non‐GWAS cohort of UKB European ancestry participants (*n* = 362,286) who were not included in the TMFI GWAS.

PRS was primarily derived using a clumping‐and‐thresholding (C + T) approach. SNPs with GWAS *p* values < 0.05 were retained, followed by LD clumping to remove correlated variants (*r* ^2^ ≥ 0.01 within a 5 Mb window). PRS computation was carried out in PLINK2 (v2.00a6LM) using the score function, which calculates the sum of risk allele counts multiplied by their corresponding GWAS effect sizes for all selected SNPs.

In addition, a Bayesian PRS was constructed as a sensitivity analysis using PRS‐CS, which integrates external LD information from the 1000 Genomes Project European reference panel (ldblk_1kg_eur) to infer posterior SNP effect sizes (Ge et al. 2019). The PRS‐CS model was implemented via its Python‐based command‐line interface (https://github.com/getian107/PRScs). Individual‐level PRSs were then computed as the weighted sum of risk allele dosages using the posterior effect estimates generated by PRS‐CS. The PRS‐CS‐derived PRS was used exclusively for association analyses with health outcomes and was not included in interaction or mediation analyses.

### 4.9 Drug Target Annotation

The DGIdb (http://www.dgidb.org/) is a comprehensive repository that consolidates multiple up‐to‐date sources of information on drugs, genes, and their interactions (including Ensembl, Drugs@FDA, and PubChem), and it catalogs over 10,000 genes and 20,000 drugs involved in more than 70,000 drug–gene interactions (Cannon et al. 2024). To explore potential therapeutic targets for TMFI, we submitted the mapped genes prioritized by the three gene‐mapping strategies to DGIdb to retrieve existing drugs and small‐molecule compounds acting on these genes. Each identified interaction was further assessed for credibility based on evidence scores compiled from authoritative data sources.

### 4.10 Cell Types and Enriched Pathways Associated With TMFI

To identify cell types implicated in TMFI, we applied the single‐cell pathway–associated genome‐wide association study (scPagwas, R version 2.0.0) in combination with the Seurat R package (version 5.1.0) to compute TRSs for TMFI across different thigh muscle cell types. scPagwas integrates GWAS summary statistics for TMFI with single‐cell transcriptomic profiles to quantify trait associations at the cellular level (Ma et al. 2023). The single‐cell transcriptomic dataset used in this analysis was derived from Lovrić et al. (2022), which included 33,975 thigh muscle cells isolated from the vastus lateralis of six human donors. Using the scPagwas R package, we profiled the distribution of TRSs across cell types and evaluated their significance, with cell types exhibiting *p* < 0.05 considered significantly associated with TMFI. We further investigated the enriched Kyoto Encyclopedia of Genes and Genomes (KEGG) pathways for each cell type. For each pathway, scPagwas was used to evaluate four parameters: the log‐rank *p* value, the normalized score, the specificity index, and the proportion of cells within each cell type genetically influenced by that pathway.

### 4.11 Summary‐Data‐Based Mendelian Randomization and TWAS

To investigate the effects of gene expression on TMFI across potentially relevant tissues—including skeletal muscle, cultured fibroblasts, adipose visceral omentum, adipose subcutaneous, lung, liver, kidney cortex, spleen, and whole blood—we integrated TMFI GWAS summary statistics with GTEx v8 eQTL data to perform summary‐data‐based Mendelian randomization (SMR) and TWAS. SMR analyses were conducted through the SMR Portal (https://yanglab.westlake.edu.cn/smr‐portal/) using eQTLs meeting a significance threshold of *p* < 5 × 10^−8^. To evaluate whether a single variant underlies both trait and gene expression associations, the heterogeneity in dependent instruments (HEIDI) test (Zhu et al. 2016) was applied using eQTLs with *p* < 1.57 × 10^−3^, the default threshold for the HEIDI test. Associations were considered significant if SMR *p* < 0.05 and HEIDI *p* > 0.05. TWAS was performed using the S‐PrediXcan (v0.8.1) approach with eQTL weights derived from MASHR models.

### 4.12 Proteomic and Metabolomic Signature for TMFI

Using proteomic and metabolomic data from the UKB, we constructed one‐dimensional convolutional neural networks (1D‐CNNs) to derive a proteomic and metabolomic signature for TMFI. The 1D‐CNN models used plasma concentrations of 2923 proteins or 251 metabolites as input features, with TMFI designated as the output variable. The 1D‐CNN architecture comprised eight convolutional layers (kernel size = 2, channels = 32, stride = 1), interleaved with three pooling layers (pooling window size = 2), where one pooling layer followed every two convolutional layers and concluded with two fully connected layers. The dataset was randomly divided into training and test sets at a 4:1 ratio. Model performance was assessed using fivefold cross‐validation. Model training was conducted using the Adam optimizer (learning rate = 1 × 10^−4^) with SELU activation functions. Weights were initialized using the LeCun normal initialization scheme. The model was trained using mean squared error (MSE) as the loss function, and early stopping was applied with a patience of 20 epochs. Model performance in both cross‐validation and holdout testing was evaluated using mean absolute error (MAE), MSE, and the Pearson correlation coefficient. All computational analyses were conducted in Python (version 3.10.16), employing TensorFlow (version 2.9.0) and Keras (version 2.9.0) for model construction and training, and scikit‐learn (version 1.6.0) for data splitting. Specifically, the protein signature was developed in 4959 UKB participants with both proteomic and TMFI MRI data at imaging visit V2, while the metabolite signature was derived in 31,991 participants with corresponding metabolomic and imaging data. For subsequent Cox regression analyses of mortality and disease outcomes, we restricted the analysis to participants with available proteomic or metabolomic data who had not undergone TMFI MRI at V2, forming independent application cohorts of 39,534 participants for the proteomic analyses and 234,082 participants for the metabolomic analyses.

### 4.13 Pathway Enrichment Analysis, Tissue Mapping, and Subcellular Localization of TMFI‐Associated Proteins

For genes corresponding to proteins significantly associated with TMFI, we conducted KEGG and GO pathway enrichment analyses using the “ClusterProfiler” R package. Enrichment was considered statistically significant at a BH‐adjusted *p* value < 0.05. To infer the likely tissue origins of these proteins, we conducted tissue enrichment analysis by mapping the identified proteins to established tissue‐specific expression profiles from the GTEx project and the Human Protein Atlas (HPA) databases (Oh et al. 2023; Carrasco‐Zanini et al. 2024). Additionally, subcellular localization enrichment analysis was carried out to characterize the cellular distribution of these proteins, classifying them as secreted, plasma membrane‐associated, or intracellular (Goeminne et al. 2025).

### 4.14 Statistical Analyses

Partial correlation analyses were performed to evaluate the associations of TMFI with eight aging‐related markers (frailty, stronger HGS, weaker HGS, reaction time, FVC, FEV1, heel BMD, and telomere length), five inflammation‐related markers (neutrophil count, lymphocyte count, monocyte count, C‐reactive protein, and SII), and parental age at death. All models were adjusted for age, sex, ethnicity, education, and the time interval between the variables.

The Cox proportional hazards regression model was employed to examine the associations of TMFI, the PRS for TMFI, the proteomic or metabolomic signature for TMFI, and various lifestyle factors with the incidences of different health outcomes. All models were adjusted for age, sex, ethnicity, and education, except for those involving the TMFI PRS, for which ethnicity was not included because analyses were restricted to individuals of European ancestry. In addition, we further adjusted for BMI in a sensitivity analysis when examining the associations between TMFI and health outcomes. Hazard ratios (HRs) with 95% confidence intervals were visualized using forest plots, while nonlinear HR trends were depicted using RCSs. Records in which the outcome occurred prior to enrollment were excluded from all survival analyses. Enrollment time was defined based on the assessment date of exposure measurements. For analyses with disease incidence as the outcome, the study end time was defined as the earliest among the date of the outcome event, the date of death, or the study termination date (October 31, 2022). For death outcomes, including all‐cause and disease‐specific mortality, the end time was defined as the date of death or the study termination date (July 8, 2024). Survival time was calculated in days from enrollment to the end time. To address potential reverse causation, we performed landmark survival analyses by introducing a 1‐year exclusion period, a 3‐year landmark, and a 5‐year landmark. In these analyses, follow‐up began at each landmark time point, and participants who experienced events prior to the respective landmark were excluded. Besides, sensitivity analyses restricted to participants who were free of all outcome diseases at V2 assessment were conducted. In addition, we further tested multiplicative interactions by incorporating product terms between TMFI and age (< 60 vs. ≥ 60 years) or sex into the Cox proportional hazards models. When the *p* value for an interaction term was < 0.05, stratified analyses were conducted to estimate the associations of TMFI with health outcomes within different age and sex groups. For system‐specific diseases, logistic regression analyses were conducted to evaluate the cross‐sectional associations between TMFI and the prevalences of these diseases, adjusting for age, sex, ethnicity, and education.

To estimate life expectancy, proportional hazards survival analyses were performed using the stpm2 command in Stata 17 MP, which models the baseline cumulative hazard via RCSs, adjusting for sex, education, and ethnicity (Chudasama et al. 2019). The calculation of life‐year loss, defined as the difference in life expectancy, involved two steps using a flexible parametric survival model with age as the time scale. First, residual life expectancy was estimated by calculating the area under the survival curve up to 100 years of age, conditional on surviving from 40 to 100 years at 1‐year intervals. Second, life‐year loss was derived by computing the difference between the areas under the survival curves for participants in the lowest TMFI quartile (1st quartile) and those in the highest TMFI quartile (4th quartile). For each of the 12 system‐specific diseases, participants were stratified into three groups: early‐onset (top 10% youngest at disease diagnosis), other‐onset (all other patients not in the top 10% youngest), and disease‐free controls (participants without a diagnosis of that specific disease). The Emmeans test was used to perform pairwise comparisons of TMFI estimated marginal means, adjusting for age as a covariate. Multiple testing for the two‐sided *p* values obtained from the Emmeans analyses was controlled using the BH method, and results with a BH‐adjusted *p* value below 0.05 were deemed statistically significant.

Analyses of lifestyle factors in relation to TMFI using RCS models were conducted with adjustments for age, sex, education, ethnicity, and the time interval between exposure and outcome collection. The optimal number of knots (*k*) for the RCS transformations was selected by minimizing the Akaike Information Criterion (AIC), with *k* set between 3 and 5 across all models.

Mediation analyses employed the product‐of‐coefficients method with 1000 bootstrap resamples to estimate mediating effects and 95% confidence intervals, while adjusting for age, sex, education, and the time interval between the V0 and the V2; ethnicity was additionally adjusted for in all mediation analyses except those involving the TMFI PRS. To specifically assess whether TMFI (measured at V2) mediates the relationship between lifestyle factors (exposures collected at V0) and the incidences of various health outcomes, we set the study start time to V2 in the Cox analyses of the exposures and outcomes and additionally adjusted for the time interval between V0 and V2 in the model. For the analyses of the associations between lifestyle factors and health outcomes, as well as the mediating role of TMFI, physical activity, BMI, and sleep duration were converted into categorical variables, whereas diet quality, smoking, and alcohol consumption were retained as their original continuous variables.

Linear regression models were used to assess how lifestyle factors, PRS, circulating plasma proteins, and plasma metabolites relate to TMFI, with adjustments made for age, ethnicity (not in the analysis of PRS), sex, education, and the V0–V2 interval. Prior to analysis, quantitative measurements of all plasma proteins and metabolites were normalized. We then evaluated both multiplicative and additive interactions between lifestyle factors and the PRS (split at the median) on TMFI. Multiplicative interactions were examined by adding PRS and each lifestyle factor product terms to the linear regression models, with statistical significance defined as a *p* value < 0.05 for the interaction term. We performed stratified analyses to evaluate how lifestyle factors influenced TMFI separately within the low‐PRS and high‐PRS groups. Next, we assessed how various combinations of PRS and lifestyle factors influenced TMFI, using individuals with low PRS and favorable lifestyle profiles (high physical activity, high diet score, normal sleep duration, non‐obesity, low alcohol consumption, and no smoking) as the reference group. Additive interaction was assessed by calculating the relative excess risk due to interaction (RERI), implemented through the “InteractionR” R package.

Statistical analyses were carried out in R (version 4.4.1), Python (version 3.10.16), or Stata (version 17). Unless stated otherwise, statistical significance was defined as a two‐tailed *p* value < 0.05.

## Author Contributions

Yan Zhang, Guangfeng Ruan, and Changhai Ding conceived the project and supervised the research; Yiwei Zhang, Qin Dang, Xizeng Zong, and Haowei Chen performed most of the data analysis; Guangfeng Ruan and Xizeng Zong provided technical support; Simin Wen, Siqi Xu, Kunyan Wang, Xiaoshuai Wang, Jianwei Zhu, and Zhaohua Zhu performed part of the data analysis and participated in the discussion; Yiwei Zhang, Guangfeng Ruan, and Qin Dang drafted the manuscript. All the authors have read and approved the final version of the manuscript.

## Funding

This work was supported by National Natural Science Foundation of China, 82472478, 82373653; Science and Technology Program of Guangzhou, China, 2025A04J3955.

## Conflicts of Interest

The authors declare no conflicts of interest.

## Supporting information


**Table S1:** Partial correlations of thigh muscle fat infiltration with aging‐related biomarkers and inflammation biomarkers.
**Table S2:** Associations between thigh muscle fat infiltration and the risk of different health outcomes.
**Table S3:** Effects of thigh muscle fat infiltration on different health outcomes across age or sex group.
**Table S4:** Distributions of marginal mean estimates of thigh muscle fat infiltration across different groups with diverse system‐specific diseases.
**Table S5:** Comparisons of marginal mean estimates of thigh muscle fat infiltration between different groups across various system‐specific diseases.
**Table S6:** Associations of lifestyle factors with mortality and the incidences of multiple diseases.
**Table S7:** Mediating effects of thigh muscle fat infiltration on the associations of lifestyle factors with mortality and the incidences of multiple diseases.
**Table S8:** Lead SNPs and genomic risk loci identified in the GWAS of thigh muscle fat infiltration.
**Table S9:** Results of genome‐wide gene‐based association study.
**Table S10:** Results of FUMA GWAS positional mapping.
**Table S11:** Results of FUMA GWAS eQTL mapping.
**Table S12:** Enrichment of mapped genes from three approaches in the MsigDB c1.
**Table S13:** Enrichment of mapped genes from three approaches in the GWAS Catalog gene sets.
**Table S14:** Associations of the polygenic risk score for thigh muscle fat infiltration with mortality and the incidences of multiple diseases in the non‐GWAS cohort.
**Table S15:** Effects of lifestyle factors on thigh muscle fat infiltration in the low‐PRS group and the high‐PRS group.
**Table S16:** Effects of different combinations of lifestyle factors and polygenic risk score on thigh muscle fat infiltration.
**Table S17:** Drug‐gene interactions for genes associated with thigh muscle fat infiltration.
**Table S18:** Trait‐relevant scores for thigh muscle cells linked to thigh muscle fat infiltration.
**Table S19:** Results of KEGG pathway enrichment analyses across trait‐relevant cell types of thigh muscle cell.
**Table S20:** Trait‐relevant scores for subtypes of myogenic cell linked to thigh muscle fat infiltration.
**Table S21:** Results of KEGG pathway enrichment analyses across trait‐relevant cell types of myogenic cell.
**Table S22:** Associations between gene expression and thigh muscle fat infiltration across tissues potentially related to the thigh muscle fat infiltration through SMR analyses.
**Table S23:** Associations between gene expression and thigh muscle fat infiltration across tissues potentially related to the thigh muscle fat infiltration through TWAS.
**Table S24:** Genes consistently identified by SMR and TWAS across tissues potentially related to the thigh muscle fat infiltration.
**Table S25:** Associations between 2923 plasma circulating proteins and thigh muscle fat infiltration.
**Table S26:** Significantly enriched GO pathways for genes of thigh muscle fat infiltration‐associated proteins.
**Table S27:** Significantly enriched KEGG pathways for genes of thigh muscle fat infiltration‐associated proteins.
**Table S28:** Associations between 251 plasma metabolites and thigh muscle fat infiltration.
**Table S29:** Mediation analysis of 2923 plasma proteins linking lifestyle factors and polygenic risk score to thigh muscle fat infiltration.
**Table S30:** Mediation analysis of 251 plasma metabolites linking lifestyle factors and polygenic risk score to thigh muscle fat infiltration.
**Table S31:** Significantly enriched GO pathways for genes of proteins significantly mediating the associations of lifestyle factors and polygenic risk score with thigh muscle fat infiltration.
**Table S32:** Significantly enriched KEGG pathways for genes of proteins significantly mediating the associations of lifestyle factors and polygenic risk score with thigh muscle fat infiltration.
**Table S33:** Evaluation metrics for the performance of the proteomic and metabolomic signature model.
**Table S34:** Associations of proteomic and metabolomic signature for thigh muscle fat infiltration with mortality and the incidences of multiple diseases.

**Figure S1:** Forest plot depicting hazard ratios for associations of thigh muscle fat infiltration with mortality and the incidences of multiple diseases. (A) Forest plot depicting hazard ratios for associations of left anterior thigh muscle fat infiltration (TMFI) with mortality and the incidences of multiple diseases. (B) Forest plot depicting hazard ratios for associations of right anterior TMFI with mortality and the incidences of multiple diseases. (C) Forest plot depicting hazard ratios for associations of left posterior TMFI with mortality and the incidences of multiple diseases. (D) Forest plot depicting hazard ratios for associations of right posterior TMFI with mortality and the incidences of multiple diseases.
**Figure S2:** Restricted cubic spline curves depicting the associations of thigh muscle fat infiltration with mortality and the incidences of multiple diseases. (A) Restricted cubic spline (RCS) curves depicting the associations of left anterior thigh muscle fat infiltration (TMFI) with mortality and the incidences of multiple diseases. (B) RCS curves depicting the associations of right anterior TMFI with mortality and the incidences of multiple diseases. (C) RCS curves depicting the associations of left posterior TMFI with mortality and the incidences of multiple diseases. (D) RCS curves depicting the associations of right posterior TMFI with mortality and the incidences of multiple diseases.
**Figure S3:** Restricted cubic spline plots depicting the associations of thigh muscle fat infiltration with mortality and the incidences of multiple diseases. The median of thigh muscle fat infiltration is used as the reference for the restricted cubic spline curves.
**Figure S4:** Forest plots depicting hazard ratios for associations between thigh muscle fat infiltration and the risk of disease‐specific mortality. (A) Forest plot depicting hazard ratios for associations of left anterior thigh muscle fat infiltration (TMFI) with disease‐specific mortality. (B) Forest plot depicting hazard ratios for associations of right anterior TMFI with disease‐specific mortality. (C) Forest plot depicting hazard ratios for associations of left posterior TMFI with disease‐specific mortality. (D) Forest plot depicting hazard ratios for associations of right posterior TMFI with disease‐specific mortality. (E) Forest plot depicting hazard ratios for associations of the total TMFI with disease‐specific mortality. (F) Forest plot depicting hazard ratios for associations of TMFI with disease‐specific mortality, estimated using a delayed‐entry design with follow‐up starting 1 year after enrollment and excluding incident cases occurring within the first year.
**Figure S5:** Forest plots depicting ratio measures for associations between thigh muscle fat infiltration and the risk of different health outcomes. (A) Forest plot depicting hazard ratios for associations of TMFI with mortality and the incidences of system‐specific diseases, estimated using a delayed‐entry design with follow‐up starting 1 year after enrollment and excluding incident cases occurring within the first year. (B) Forest plot depicting hazard ratios for associations of TMFI with mortality and the incidences of system‐specific diseases, estimated using a delayed‐entry design with follow‐up starting 3 years after enrollment and excluding incident cases occurring within the 3 years. (C) Forest plot depicting hazard ratios for associations of TMFI with mortality and the incidences of system‐specific diseases, estimated using a delayed‐entry design with follow‐up starting 5 years after enrollment and excluding incident cases occurring within the 5 years. (D) Forest plot depicting hazard ratios for associations of TMFI with mortality and the incidences of system‐specific diseases, with participants having preexisting any of the outcome diseases at V2 excluded from the analyses. (E) Forest plot depicting hazard ratios for associations of TMFI with mortality and the incidences of system‐specific diseases, with additional adjustment for body mass index (BMI). (F) Forest plot depicting odds ratios for associations between TMFI and the prevalences of system‐specific diseases.
**Figure S6:** Effects of thigh muscle fat infiltration on different health outcomes across age and sex group. *p* indicate the statistical multiplicative interaction of age or sex with TMFI on the different health outcomes. Bar heights represent the hazard ratios of TMFI on the different health outcomes in different groups. Asterisk indicates significant association between TMFI and the health outcomes in the corresponding group. **p value < 0.01, ***p value < 0.001.
**Figure S7:** Forest plots depicting hazard ratios for associations of lifestyle factors with mortality and the incidences of multiple diseases. Forest plots depicting hazard ratios for associations of lifestyle exposures with mortality and the incidences of system‐specific diseases.
**Figure S8:** Post‐GWAS functional characterization of thigh muscle fat infiltration. (A) Q‐Q plot of the genome‐wide association study (GWAS) for thigh muscle fat infiltration (TMFI). The *x*‐axis and *y*‐axis represent the negative base‐10 logarithm of the expected theoretical *p* values and the observed *p* values, respectively. (B) Functional consequences of candidate single nucleotide polymorphisms (SNPs) on genes. The histogram displays the proportion of candidate SNPs (linkage disequilibrium *r* ^2^ ≥ 0.6 of any independent significant SNPs and GWAS *p* < 0.05) that have corresponding functional annotation assigned by Annotation of Variants Using Next‐generation ARray and VARIation (ANNOVAR). Bar color represents the enrichment value, which is calculated as the proportion of annotated candidate SNPs divided by the proportion of annotated SNPs in the reference panel. Fisher's exact test (two‐sided) is performed for each annotation. *p < 0.05, **p < 0.05/11. (C) Multidimensional annotations of genomic risk loci based on GWAS of TMFI. Genomic risk loci are displayed in the format of “chromosome: start position ‐ end position” on the *y*‐axis. Bar length from the leftmost to the rightmost figure represents the size of the genomic loci, the number of candidate SNPs within the loci, the number of genes physically located in the loci, and the number of mapped genes identified through positional mapping, and expression quantitative trait locus (eQTL) mapping, respectively. (D) Multi‐marker Analysis of GenoMic Annotation (MAGMA) gene property analysis in 54 specific tissue types from Genotype‐Tissue Expression (GTEx) v8 shows the enrichment of TMFI‐associated genes in nine tissue types. Corresponding tissue names are indicated below the bars. Bar height depicts −log_10_ *p* value from one‐sided *t*‐test for each enrichment, and the dotted horizontal line represents the statistical significance (*p* = 0.05).
**Figure S9:** PRS‐CS–derived polygenic risk score for thigh muscle fat infiltration and risk of mortality and disease incidence. Forest plot depicting hazard ratios for associations of the polygenic risk score for thigh muscle fat infiltration, constructed using the PRS‐CS, with mortality and the incidences of multiple diseases in the non‐GWAS cohort.
**Figure S10:** Interactions Between Polygenic Risk Score and Lifestyle Factors on Thigh Muscle Fat Infiltration. (A) Effects of lifestyle factors on thigh muscle fat infiltration (TMFI) in the low‐polygenic risk score (PRS) group and the high‐PRS group. *p*‐interactions indicate the statistical multiplicative interactions between lifestyle factors and PRS that affect TMFI. Bar heights represent the odds ratios of lifestyle factors on TMFI in different groups. (B) Heatmap depicting the effects of different combinations of lifestyle factors and PRS on TMFI. The color gradient indicates the odds ratios of different combinations of lifestyle factors and PRS on TMFI. The reference was set as the combination pattern in the top‐left cell of the heatmap. Numeric values within the boxes represent the relative excess risk due to interaction (RERI). PRS, polygenic risk score. The PRS was calculated using the clumping‐and‐thresholding (C + T) method. Participants were stratified into low and high groups based on the median values of PRS, physical activity, diet score, and alcohol consumption, respectively. Obesity: BMI ≥ 30 kg/m^2^; non‐obesity: BMI < 30 kg/m^2^. Long sleep duration: duration ≥ 8 h/day; normal sleep duration: 6 h ≤ duration < 8 h/day; short sleep duration: duration < 6 h/day. The reference for long sleep duration and short sleep duration is normal sleep duration. Smoking: pack years of smoking > 0; no smoking: pack years of smoking = 0. *p < 0.05.
**Figure S11:** Cell markers and single‐cell trait‐relevant scores (TRSs) for thigh muscle fat infiltration. (A) Marker gene expressions of different cell types in skeletal muscle. The color intensity of the circle indicates the average of standardized gene expression levels within each cell type, and the size of the circle indicates the percentage of cells expressing the given gene within each cell type. (B) Single‐cell trait‐relevant scores (TRSs) for thigh muscle fat infiltration (TMFI) of thigh muscle cells computed via single‐cell pathway‐associated genome‐wide association study (scPagwas) analysis within the Seurat framework. (C) Marker gene expressions of different cell types in myogenic cell. The color intensity of the circle indicates the average of standardized gene expression levels within each cell subtype, and the size of the circle indicates the percentage of cells expressing the given gene within each cell subtype. (D) Single‐cell TRSs for TMFI of myogenic cells computed via scPagwas analysis within the Seurat framework.
**Figure S12:** Upset plots depicting genes identified by (A) summary‐data‐based mendelian randomization or (B) Transcriptome‐wide association study with intersection analysis. The horizontal bars on the left represent the number of genes identified in each of the nine tissues potentially related to the thigh muscle fat infiltration (TMFI) by the respective method. Dots and connecting lines indicate combinations of tissues potentially related to the TMFI. Vertical bars represent the intersection size (number of overlapping genes) for each combination. For individual dots (without connections), the corresponding vertical bar denotes the count of tissue‐specific genes.
**Figure S13:** Tissue expression mapping for thigh muscle fat infiltration‐associated proteins based on (A) the genotype‐tissue expression (GTEx) or (B) the human protein atlas (HPA) databases. Bar color indicates the subcellular localization of thigh muscle fat infiltration‐associated proteins. Bar length represents the number of mapped proteins in each tissue. GTEx, Genotype‐Tissue Expression; HPA, Human Protein Atlas.
**Figure S14:** Bubble plot depicting plasma circulating proteins significantly mediating the associations between lifestyle factors and thigh muscle fat infiltration. Bubble color indicates standardized coefficient from linear regression analysis of the effect of the plasma proteins on thigh muscle fat infiltration, while bubble size reflects the mediation proportion. The top 20 proteins with the highest mediation proportions are presented.
**Figure S15:** Bubble plot depicting plasma metabolites significantly mediating the associations between lifestyle factors and thigh muscle fat infiltration. Bubble color indicates standardized coefficient from linear regression analysis of the effect of the plasma metabolites on thigh muscle fat infiltration, while bubble size reflects the mediation proportion. The top 20 metabolites with the highest mediation proportions are presented.
**Figure S16:** Top enriched pathways, tissue expression mapping, and subcellular localization for mediator proteins. Mediator proteins included in the analyses are those that significantly mediate the effect of polygenic risk score (PRS) and lifestyle factors on thigh muscle fat infiltration. Left panels depict the top significantly enriched Gene Ontology (GO) and Kyoto Encyclopedia of Genes and Genomes (KEGG) pathways. Bar length represents the statistical significance of enrichment. The size of the bubbles and the numbers indicate the count of enriched genes. The middle and right panels represent tissue expression mapping for mediator proteins using data from the Genotype‐Tissue Expression (GTEx) or the Human Protein Atlas (HPA) databases. Bar color indicates the subcellular localization of mediator proteins. Bar length represents the number of mapped proteins in each tissue. The PRS was calculated using the clumping‐and‐thresholding (C + T) method. GO, gene ontology; GTEx, genotype‐tissue expression; HPA, human protein atlas; KEGG, Kyoto Encyclopedia of Genes and Genomes.

## Data Availability

This research has been conducted using data from the UK Biobank under approved Application Number 131591. Single‐cell sequencing data can be found in GEO at https://www.ncbi.nlm.nih.gov/geo/ under the accession number GSE214544. The genome‐wide association study (GWAS) summary statistics generated in this study have been deposited in Zenodo and are publicly available at https://doi.org/10.5281/zenodo.21017350. The analysis code used in this study is available on GitHub at https://github.com/zyw‐x/Zhang.
