# Supplementary material for: Integrative Analysis Uncover the Effects and Multi‐Omics Features of Thigh Muscle Fat Infiltration
Source: Aging Cell. 2026 Aug 31;25(9):e70690. doi: 10.1111/acel.70690 (PMC13530289; doi:10.1111/acel.70690)
Supplement: Supplementary file 1 [file ACEL-25-e70690-s001.docx]

**Figure S1. Forest plot depicting hazard ratios for associations of Thigh Muscle Fat Infiltration with mortality and the incidences of multiple diseases. (A)** Forest plot depicting hazard ratios for associations of left anterior thigh muscle fat infiltration (TMFI) with mortality and the incidences of multiple diseases. **(B)** Forest plot depicting hazard ratios for associations of right anterior TMFI with mortality and the incidences of multiple diseases. **(C)** Forest plot depicting hazard ratios for associations of left posterior TMFI with mortality and the incidences of multiple diseases. **(D)** Forest plot depicting hazard ratios for associations of right posterior TMFI with mortality and the incidences of multiple diseases.

**Figure S2. Restricted Cubic Spline Curves Depicting the Associations of** **Thigh Muscle Fat Infiltration with Mortality and the Incidences of Multiple Diseases.**

**(A)** Restricted cubic spline (RCS) curves depicting the associations of left anterior thigh muscle fat infiltration (TMFI) with mortality and the incidences of multiple diseases. **(B)** RCS curves depicting the associations of right anterior TMFI with mortality and the incidences of multiple diseases. **(C)** RCS curves depicting the associations of left posterior TMFI with mortality and the incidences of multiple diseases. **(D)** RCS curves depicting the associations of right posterior TMFI with mortality and the incidences of multiple diseases.

**Figure S3. Restricted Cubic Spline Plots Depicting the Associations of Thigh Muscle Fat Infiltration with Mortality and the Incidences of Multiple Diseases.** The median of thigh muscle fat infiltration is used as the reference for the restricted cubic spline curves.

**Figure S4. Forest Plots Depicting Hazard Ratios for Associations Between Thigh Muscle Fat Infiltration and the Risk of Disease-Specific Mortality. (A)** Forest plot depicting hazard ratios for associations of left anterior thigh muscle fat infiltration (TMFI) with disease-specific mortality. **(B)** Forest plot depicting hazard ratios for associations of right anterior TMFI with disease-specific mortality. **(C)** Forest plot depicting hazard ratios for associations of left posterior TMFI with disease-specific mortality. **(D)** Forest plot depicting hazard ratios for associations of right posterior TMFI with disease-specific mortality. **(E)** Forest plot depicting hazard ratios for associations of the total TMFI with disease-specific mortality. **(F)** Forest plot depicting hazard ratios for associations of TMFI with disease-specific mortality, estimated using a delayed-entry design with follow-up starting one year after enrollment and excluding incident cases occurring within the first year.

**Figure S5. Forest Plots Depicting Ratio Measures for Associations Between Thigh Muscle Fat Infiltration and the Risk of Different Health Outcomes. (A)** Forest plot depicting hazard ratios for associations of TMFI with mortality and the incidences of system-specific diseases, estimated using a delayed-entry design with follow-up starting one year after enrollment and excluding incident cases occurring within the first year. **(B)** Forest plot depicting hazard ratios for associations of TMFI with mortality and the incidences of system-specific diseases, estimated using a delayed-entry design with follow-up starting three years after enrollment and excluding incident cases occurring within the three years. **(C)** Forest plot depicting hazard ratios for associations of TMFI with mortality and the incidences of system-specific diseases, estimated using a delayed-entry design with follow-up starting five years after enrollment and excluding incident cases occurring within the five years. **(D)** Forest plot depicting hazard ratios for associations of TMFI with mortality and the incidences of system-specific diseases, with participants having pre-existing any of the outcome diseases at V2 excluded from the analyses. **(E)** Forest plot depicting hazard ratios for associations of TMFI with mortality and the incidences of system-specific diseases, with additional adjustment for body mass index (BMI). **(F)** Forest plot depicting odds ratios for associations between TMFI and the prevalences of system-specific diseases.

**Figure S6. Effects of Thigh Muscle Fat Infiltration on Different Health Outcomes across Age and Sex Group.** *P* indicate the statistical multiplicative interaction of age or sex with TMFI on the different health outcomes. Bar heights represent the hazard ratios of TMFI on the different health outcomes in different groups. Asterisk indicates significant association between TMFI and the health outcomes in the corresponding group. **: *P* value < 0.01, ***: *P* value < 0.001.

**Figure S7. Forest Plots Depicting Hazard Ratios for Associations of Lifestyle Factors with Mortality and the Incidences of Multiple Diseases.** Forest plots depicting hazard ratios for associations of lifestyle exposures with mortality and the incidences of system-specific diseases.

**Figure S8. Post-GWAS Functional Characterization of** **Thigh Muscle Fat Infiltration. (A)** Q-Q plot of the genome-wide association study (GWAS) for thigh muscle fat infiltration (TMFI). The x-axis and y-axis represent the negative base-10 logarithm of the expected theoretical *P* values and the observed *P* values, respectively. **(B)** Functional consequences of candidate single nucleotide polymorphisms (SNPs) on genes. The histogram displays the proportion of candidate SNPs (linkage disequilibrium r² ≥ 0.6 of any independent significant SNPs and GWAS *P* < 0.05) that have corresponding functional annotation assigned by Annotation of Variants Using Next-generation ARray and VARIation (ANNOVAR). Bar color represents the enrichment value, which is calculated as the proportion of annotated candidate SNPs divided by the proportion of annotated SNPs in the reference panel. Fisher's exact test (two-sided) is performed for each annotation. *: *P* < 0.05, **: *P* < 0.05/11. **(C)** Multi-dimensional annotations of genomic risk loci based on GWAS of TMFI. Genomic risk loci are displayed in the format of "chromosome: start position - end position" on the y-axis. Bar length from the leftmost to the rightmost figure represents the size of the genomic loci, the number of candidate SNPs within the loci, the number of genes physically located in the loci, and the number of mapped genes identified through positional mapping, and expression quantitative trait locus (eQTL) mapping, respectively. **(D)** Multi-marker Analysis of GenoMic Annotation (MAGMA) gene property analysis in 54 specific tissue types from Genotype-Tissue Expression (GTEx) v8 shows the enrichment of TMFI-associated genes in nine tissue types. Corresponding tissue names are indicated below the bars. Bar height depicts -log_10_ *P* value from one-sided t-test for each enrichment, and the dotted horizontal line represents the statistical significance (*P* = 0.05).

**Figure S9. PRS-CS–Derived Polygenic Risk Score for Thigh Muscle Fat Infiltration and Risk of Mortality and Disease Incidence****.** Forest plot depicting hazard ratios for associations of the polygenic risk score for thigh muscle fat infiltration, constructed using the PRS-CS, with mortality and the incidences of multiple diseases in the non-GWAS cohort.

**Figure S10. Interactions Between Polygenic Risk Score and Lifestyle Factors on** **Thigh Muscle Fat Infiltration. (A)** Effects of lifestyle factors on thigh muscle fat infiltration (TMFI) in the low-polygenic risk score (PRS) group and the high-PRS group. *P*-interactions indicate the statistical multiplicative interactions between lifestyle factors and PRS that affect TMFI. Bar heights represent the odds ratios of lifestyle factors on TMFI in different groups. **(B)** Heatmap depicting the effects of different combinations of lifestyle factors and PRS on TMFI. The color gradient indicates the odds ratios of different combinations of lifestyle factors and PRS on TMFI. The reference was set as the combination pattern in the top-left cell of the heatmap. Numeric values within the boxes represent the relative excess risk due to interaction (RERI). **Abbreviation:** PRS, polygenic risk score. **Notes:** The PRS was calculated using the clumping-and-thresholding (C+T) method. Participants were stratified into low and high groups based on the median values of PRS, physical activity, diet score, and alcohol consumption, respectively. Obesity: BMI ≥ 30 kg/m^2^; non-obesity: BMI < 30 kg/m^2^. Long sleep duration: duration ≥ 8 hours/day; normal sleep duration: 6 hours ≤ duration < 8 hours/day; short sleep duration: duration < 6 hours/day. The reference for long sleep duration and short sleep duration is normal sleep duration. Smoking: pack years of smoking > 0; no smoking: pack years of smoking = 0. *: *P* < 0.05.

**Figure S11. Cell Markers and Single-Cell Trait-Relevant Scores (TRSs) for** **Thigh Muscle Fat Infiltration. (A)** Marker gene expressions of different cell types in skeletal muscle. The color intensity of the circle indicates the average of standardized gene expression levels within each cell type, and the size of the circle indicates the percentage of cells expressing the given gene within each cell type. **(B)** Single-cell trait-relevant scores (TRSs) for thigh muscle fat infiltration (TMFI) of thigh muscle cells computed via single-cell pathway-associated genome-wide association study (scPagwas) analysis within the Seurat framework. **(C)** Marker gene expressions of different cell types in myogenic cell. The color intensity of the circle indicates the average of standardized gene expression levels within each cell sub-type, and the size of the circle indicates the percentage of cells expressing the given gene within each cell sub-type. **(D)** Single-cell TRSs for TMFI of myogenic cells computed via scPagwas analysis within the Seurat framework.

**Figure S12. Upset Plots Depicting Genes Identified by (A) Summary-data-based Mendelian Randomization or (B) Transcriptome-wide Association Study with Intersection Analysis.** The horizontal bars on the left represent the number of genes identified in each of the 9 tissues potentially related to the thigh muscle fat infiltration (TMFI) by the respective method. Dots and connecting lines indicate combinations of tissues potentially related to the TMFI. Vertical bars represent the intersection size (number of overlapping genes) for each combination. For individual dots (without connections), the corresponding vertical bar denotes the count of tissue-specific genes.

**Figure S13. Tissue Expression Mapping for** **Thigh Muscle Fat Infiltration-associated Proteins based on (A) the Genotype-Tissue Expression (GTEx) or (B) the Human Protein Atlas (HPA) databases.** Bar color indicates the subcellular localization of thigh muscle fat infiltration-associated proteins. Bar length represents the number of mapped proteins in each tissue. **Abbreviation:** GTEx, Genotype-Tissue Expression; HPA, Human Protein Atlas.

**Figure S14. Bubble Plot Depicting Plasma Circulating Proteins Significantly Mediating the Associations Between Lifestyle Factors and** **Thigh Muscle Fat Infiltration.** Bubble color indicates standardized coefficient from linear regression analysis of the effect of the plasma proteins on thigh muscle fat infiltration, while bubble size reflects the mediation proportion. The top 20 proteins with the highest mediation proportions are presented.

**Figure S15. Bubble Plot Depicting Plasma Metabolites Significantly Mediating the Associations Between Lifestyle Factors and** **Thigh Muscle Fat Infiltration.** Bubble color indicates standardized coefficient from linear regression analysis of the effect of the plasma metabolites on thigh muscle fat infiltration, while bubble size reflects the mediation proportion. The top 20 metabolites with the highest mediation proportions are presented.

**Figure S16. Top Enriched Pathways, Tissue Expression Mapping, and Subcellular Localization for Mediator Proteins.** Mediator proteins included in the analyses are those that significantly mediate the effect of polygenic risk score (PRS) and lifestyle factors on thigh muscle fat infiltration. Left panels depict the top significantly enriched Gene Ontology (GO) and Kyoto Encyclopedia of Genes and Genomes (KEGG) pathways. Bar length represents the statistical significance of enrichment. The size of the bubbles and the numbers indicate the count of enriched genes. The middle and right panels represent tissue expression mapping for mediator proteins using data from the Genotype-Tissue Expression (GTEx) or the Human Protein Atlas (HPA) databases. Bar color indicates the subcellular localization of mediator proteins. Bar length represents the number of mapped proteins in each tissue. **Note:** The PRS was calculated using the clumping-and-thresholding (C+T) method. **Abbreviations:** GTEx, Genotype-Tissue Expression; HPA, Human Protein Atlas; GO, Gene Ontology; KEGG, Kyoto Encyclopedia of Genes and Genomes.
